# The role of relationship beliefs in predicting levels and changes of relationship satisfaction

**DOI:** 10.1177/08902070241240029

**Published:** 2024-04-09

**Authors:** Fabian Gander, Maximiliane Uhlich, Alex Christoph Traut, Marcelle Ariane Saameli, Janina Larissa Bühler, Rebekka Weidmann, Alexander Grob

**Affiliations:** 1 Department of Psychology, 27209 University of Basel, Basel, Switzerland; 2 Department of Psychology, Johannes Gutenberg University Mainz, Mainz, Germany; 3 Department of Psychology, Michigan State University, East Lansing, MI, USA

**Keywords:** relationship satisfaction, implicit relationship theories, growth beliefs, destiny beliefs, romantic relationships

## Abstract

Relationship beliefs (i.e., destiny and growth beliefs) are associated with important relationship outcomes. Destiny beliefs describe the belief that a relationship is meant to be while growth beliefs describe the tendency that relationships can be cultivated and maintained through effort. Based on a longitudinal sample of people in romantic relationships (*N* = 904 couples), we examined whether destiny and growth beliefs predict current levels and trajectories of relationship satisfaction across 2 years. Using dyadic growth curve models, we found that individuals with stronger destiny beliefs generally reported higher initial relationship satisfaction. Furthermore, those with higher growth beliefs experienced a slower decline in relationship satisfaction over time. Vice versa, higher relationship satisfaction also predicted increases in growth beliefs, but not destiny beliefs over time. These findings were also supported when directly asking participants about their subjectively perceived trajectories of relationship satisfaction: Growth beliefs, but not destiny beliefs, went along with the perception that relationship satisfaction has increased in the past and will further increase in the future. The findings suggest that relationship beliefs are relevant for long-term outcomes and could have important implications for developing interventions to help couples maintain relationship satisfaction in the long term.

## Plain language summary

In relationships, relationship satisfaction tends to decrease over time, but what we believe about love can shape our satisfaction. This study of over 900 couples looked at two kinds of beliefs: “destiny beliefs” (love is meant to be) and “growth beliefs” (love grows with effort). We found that those who believe in destiny generally feel more satisfied at the start. However, people who think love grows with effort tend to see less decline in their satisfaction over 2 years. Interestingly, more satisfied couples tend to develop stronger growth beliefs over time. This suggests that our beliefs about love not only reflect our relationship satisfaction but also influence its future course. Understanding these beliefs could help us develop ways to keep couples happy together in the long run.

## Introduction

Beliefs about oneself, one’s future, or the world play a crucial role in people’s lives. This has mainly been found for depression (Beck, 1970), but also many other aspects of life, including achievement, relationships (along with sexuality; Maxwell et al., 2017), and well-being (Clifton et al., 2019; Dweck, 1999). Dweck (1999) introduced the distinction between fixed mindsets, the belief that personal characteristics are static and unchangeable, and growth mindsets, the belief that characteristics can be developed and fostered.

This distinction has also been applied to romantic relationships. In this domain, two different beliefs are distinguished which are also called implicit relationship theories: destiny beliefs assume that relationships are either meant to be or not meant to be while growth beliefs refer to the belief that relationships can be cultivated and developed (Knee, 1998; see also Franiuk et al., 2002; Sprecher & Metts, 1989, for similar approaches). Growth and destiny beliefs are associated with various desirable outcomes in romantic relationships, such as higher relationship satisfaction and commitment (Franiuk et al., 2004; Knee et al., 2004). Furthermore, growth and destiny beliefs have profound implications for relationship maintenance (e.g., Knee, 1998; Knee et al., 2004).

Given the malleability of beliefs (e.g., Yeager & Dweck, 2020), relationship beliefs might offer a promising starting point for interventions. As relationship satisfaction tends to decline over the course of a relationship (Bühler et al., 2021a), factors that predict trajectories of relationship satisfaction could make an important contribution to understanding and supporting happy relationships. However, little research has addressed whether relationship beliefs also contribute to the development of relationship satisfaction in the long term using longitudinal data. Overall, the research so far has been limited in some regards. First, many studies relied on cross-sectional data based on between-subject design or only considered shorter time intervals (e.g., less than one year). Using dyadic longitudinal data on the other hand allows tracking within-person changes (e.g., Uhlich et al., 2021) over several years which is crucial for understanding how relationship satisfaction develops over time. Second, many studies focused on student samples, which limits the conclusions to relationships among young people and those with a relatively short duration, and to a particular social context. Third, few studies examined both partners in a couple and thereby also took the partner’s beliefs into account instead of relying only on the perspective of one partner of a dyad.

The present study aims at filling these gaps by examining whether and how growth and destiny beliefs relate to current levels and changes of relationship satisfaction over the course of 2 years in a dyadic, age-heterogenous couple sample.

### Relationship beliefs

Knee (1998) distinguishes between two types of relationship beliefs, destiny beliefs and growth beliefs. *Destiny beliefs* (similar terms are entity beliefs and soulmate theories) describe the tendency to think that relationships are either meant to be or not and that the success of a relationship is mostly determined by factors outside of the partners’ control. Individuals with higher scores in destiny beliefs see relationships as more fixed and believe that challenges are unlikely to be overcome, no matter how much effort is put in. *Growth beliefs* (similar terms are incremental beliefs and work-it-out theories) describe the tendency to think that relationships can be maintained through work and grow over time. Individuals scoring high in growth beliefs see relationships as more dynamic and believe that challenges can be overcome through effort.

Relationship beliefs differ from other beliefs (e.g., mindsets regarding intelligence) in three ways: First, outside of a relationship context incremental (i.e., growth) beliefs are usually considered to be more favorable and entity (i.e., destiny) beliefs are considered less favorable (e.g., Dweck, 1999). Nevertheless, both—growth and destiny beliefs of relationships—can be adaptive and are associated with beneficial outcomes under certain circumstances (e.g., Franiuk et al., 2002; Knee et al., 2003; Weigel et al., 2016). However, people holding destiny beliefs may be more likely to engage in passive and avoidant coping strategies, for instance, avoiding problematic issues (Knee, 1998). People with growth beliefs tend to be more likely to engage in continued communication and problem-solving behaviors (Knee, 1998; Knee et al., 2003) which is especially important under stressful circumstances (Dorrance-Hall et al., 2023). This suggests that on average, couples with growth beliefs engage in more adaptive behavior compared to couples holding destiny beliefs.

Second, while beliefs in other areas are often conceptualized as one dimension with two opposing poles, relationship beliefs are conceptualized in two theoretically and statistically independent dimensions (see e.g., Knee & Canevello, 2006; Maxwell et al., 2017) that do not correlate (Knee, 1998; but see Franiuk et al., 2002 for a negative correlation).

Third, Knee (1998) showed that destiny and growth beliefs are widely independent from classical personality traits, such as the dimensions of the Five-Factor Model (“Big Five”; Goldberg, 1993) underlining that relationship beliefs capture a different construct that is not explained by personality traits. Consequently, these beliefs may contribute additional insights and explanations for variations in relationship outcomes.

### Relationship beliefs and relationship outcomes

Several studies examined the associations of relationship beliefs with relationship outcomes. In a meta-analysis on predictors of relationship dissolution, Le et al. (2010) reported a small effect of destiny beliefs as a predictor of relationship breakup while the protective effect of growth beliefs did not reach significance, probably due to large heterogeneity in the samples. A recent study by Joel et al. (2020), employing machine learning techniques to predict relationship quality, examined a large number of potential predictors and supported, among other factors, the relevance of both growth and destiny beliefs in the prediction of relationship satisfaction. However, the study by Joel et al. (2020) does not provide information on the direction of effects regarding relationship beliefs and satisfaction.

Other studies found that people higher in destiny beliefs are more satisfied and less likely to separate from their partner if they perceive their partner as “the one” (Franiuk et al., 2002; Knee, 1998). In addition to the global implications for relationship stability, further findings suggest that relationship beliefs relate to whether people interact adaptively or maladaptively in their relationships. For example, people who hold growth beliefs are more likely to forgive their partner for minor offenses (Burnette & Franiuk, 2010; Thompson et al., 2020) and are less likely to engage in intimate partner violence (Cobb et al., 2013).

Several studies identified relationship beliefs to be relevant moderators for a broad array of outcomes: Knee et al. (2004) reported a weaker association between conflict and higher commitment for those with higher growth beliefs. More specifically, even if a conflict is unresolved, individuals reporting growth beliefs stay committed. On the other hand, individuals higher in destiny beliefs interpret conflicts as a sign of incompatibility (Burnette & Franiuk, 2010; Knee, 1998; Knee et al., 2003). A recent study suggested that growth beliefs buffer against the negative impact of criticism within the couple on relationship satisfaction (Figueroa et al., 2022). Further, several studies suggested that the effects of relationship beliefs depend on whether one considers their current partner to be ideal: Franiuk et al. (2002, 2004) reported that destiny beliefs may be more beneficial when one considers one’s current partner to be ideal (i.e., the “soulmate”), while growth beliefs may be associated with greater satisfaction when one’s partner seems less than ideal. Similarly, Knee et al. (2001) demonstrated that those with a combination of high growth and low destiny beliefs are more accepting of partner imperfections, such that their relationship satisfaction is not diminished by discrepancies between the current and ideal partner. Mattingly et al. (2019) examined potential mechanisms and suggested that growth beliefs promote relationship satisfaction and prevent dissolution considerations through self-expansion, that is, being motivated to increase one’s self-concept through engagement in novel and interesting activities (Mattingly et al., 2019).

### Development of relationship satisfaction

Cross-sectional studies often report a U-shaped association between age and relationship satisfaction, with a decline in young adulthood reaching the low point around age 40, followed by an increase until retirement age (e.g., Gilford & Bengtson, 1979). Similar findings were reported for relationship duration and satisfaction: A decrease in the first 10 years of a relationship, followed by an increase for the next decade, and a following decrease and stabilization afterward (Anderson et al., 1983; Glenn, 1990). A recent meta-analysis examining longitudinal studies, however, suggested a general decrease in relationship satisfaction, with the strongest decreases for younger people and younger relationships (Bühler et al., 2021a). This is highly relevant since low relationship satisfaction predicts relationship instability and divorce (Karney & Bradbury, 1995). Relationship dissolution and separation in turn predict a number of negative outcomes such as emotional distress (Frazier & Cook, 1993; Sbarra & Emery, 2005), losing friends and social support due to social network changes (Greif & Deal, 2012), as well as poorer physical and psychological well-being (Holt-Lunstad et al., 2010; Rhoades et al., 2011).

Given this trend of deteriorating relationship satisfaction, exploring potential factors that might help to prevent this decline seems highly relevant, especially from an applied perspective. At the same time, while relevant moderators of relationship satisfaction levels, such as the presence of children, were identified, few moderators of relationship satisfaction trajectories were identified (Bühler et al., 2021a). A recent meta-analytic machine learning approach considered a broad array of predictors (Joel et al., 2020) and suggested that while the *levels* of relationship quality can be relatively well predicted (around 45% explained variance) by self-ratings of how partners are perceived and appreciated, none of the variables studied predicted the *trajectory* of relationship quality. Even though the search for potential moderators has hitherto yielded discouraging findings, it is important to consider further variables that might shape relationship quality over time.

### Partner effects of relationship beliefs

Only a small number of studies have used dyadic samples and to the best of our knowledge only one study examined partner effects of relationship beliefs in the domain of sexual beliefs: Maxwell et al. (2017) found that individuals with more pronounced sexual growth beliefs not only experience higher personal relationship and sexual satisfaction, but their partners also report greater satisfaction in both the relationship and their sex life. Regarding sexual destiny beliefs, the study observed only actor effects, which depended on the perceived compatibility with their partner: Individuals with stronger sexual destiny beliefs experienced lower relationship satisfaction when they encountered more sexual disagreements or perceived their partner as sexually less ideal. Thus, these findings suggest that the partner’s beliefs can also be relevant for relationship outcomes.

### The present study

The present study uses a longitudinal dyadic sample (i.e., four measurement waves across 2 years) of romantic couples and addresses six research questions:

RQ1 First, based on earlier research suggesting that growth and destiny beliefs predict levels of relationship satisfaction (Bőthe et al., 2017; Joel et al., 2020; Maxwell et al., 2017), we examine whether relationship beliefs (i.e., growth and destiny beliefs) predict current levels of relationship satisfaction. Here, we aimed at replicating previous findings and hypothesized that both growth and destiny beliefs predict higher current levels of relationship satisfaction.

RQ2 Second, previous studies reported that growth and destiny beliefs predict relationship dissolution (Le et al., 2010) which suggests that relationship beliefs exert long-term consequences for the stability of a relationship, potentially by leading to decreases in relationship satisfaction. Thus, we examine whether growth and destiny beliefs predict changes in relationship satisfaction over 2 years. More specifically, we study whether the changes (within-person effects) in relationship satisfaction over time are moderated by baseline levels of growth and destiny beliefs. Here, we hypothesize that especially growth beliefs predict less steep declines in relationship satisfaction over time.

RQ3 Third, if relationship beliefs shape long-term trajectories of relationship satisfaction, this should also be reflected in associations with relationship duration. Therefore, we examine whether the association between relationship duration and relationship satisfaction levels is moderated by baseline levels of growth and destiny beliefs. Here, we hypothesize that the negative association between relationship duration and relationship satisfaction is weaker for those with stronger growth beliefs.

RQ4 Fourth, as a robustness check, we also examine the results of research questions 1–3 when additionally including several individual difference constructs, namely, the Big Five dimensions of personality (see also Soto, 2019), self-esteem, satisfaction with life, and attachment orientations. We selected these constructs because they were among the most potent predictors of relationship satisfaction based on Joel and colleagues’ (2020) analyses, and they were also available in the dataset used for this study (see below). Other important predictors, such as depression, anxiety, or positive and negative affect, were not assessed at baseline in the current dataset. Thus, these additional analyses serve to examine whether relationship beliefs can predict levels and trajectories of relationship satisfaction beyond the influence of relevant individual difference constructs, or whether one of these constructs could explain potential relationships between relationship beliefs and relationship satisfaction.

RQ5 Fifth, as an exploratory research question we examine a) subjective retrospection of the past and b) expectations of future changes in relationship satisfaction using graphical illustrations (see the Methods section for more details) and how that relates to relationship beliefs. Thus, we analyze how growth and destiny beliefs relate to subjective retrospective and prospective trajectories of relationship satisfaction in addition to their changes in reported relationship satisfaction measured across 2 years. The first part of this research question served to further corroborate the results of research question 3 using a different methodology. That is, does asking participants directly about their (past) trajectories yield the same results than when assessing relationship satisfaction repeatedly and then computing the trajectories (as in research question 3)? The second part of this research question served to examine whether relationship beliefs also go along with a more positive outlook on the future development of the relationship.

For research questions 1 to 3, we examine the effects of both partners’ beliefs on relationship satisfaction, as previous research suggests actor effects (i.e., the association of one’s own relationship beliefs with one’s own subjective change trajectories) and partner effects (i.e., the association of the partner’s relationship theories with one’s own subjective change trajectories; Maxwell et al., 2017). In research question 5, we focus exclusively on actor effects.^1^

RQ6 Finally, we examined the reverse direction of research questions 1–3, namely, whether baseline levels of own and the partner’s relationship satisfaction predict the levels and trajectories of growth and destiny beliefs across time and relationship duration. The purpose of this research question was to examine if the effects are unidirectional (i.e., relationship beliefs predicting changes in relationship satisfaction) or bidirectional (i.e., relationship satisfaction also predicts changes in relationship beliefs).

## Method

We report how we determined our sample size, all data exclusions, and all measures in the study. This study was not preregistered. The data are available upon request from https://doi.org/10.48573/eben-4q86. The analysis code and supplementary tables and figures can be found on OSF (https://osf.io/3fp8j/), as well as a detailed study description and all variables (https://osf.io/m5bwj/).

### Procedure

The data for this study came from a larger project entitled *Processes in Romantic Relationships and Their Impact on Relationship and Personal Outcomes (CouPers)*^2^ examining processes in romantic relationships conducted at the University of Basel, Switzerland, between 2016 and 2018. A detailed study description and all variables in the project can be found on OSF (https://osf.io/m5bwj/), and the raw data can be requested at https://doi.org/10.48573/eben-4q86. The project surveyed romantic couples across four waves in 4- to 6-month intervals between waves 1 and 3, and a longer interval of 10–12 months between waves 3 and 4. Each wave consisted of a longer survey on each partner’s experiences in general (“trait characteristics”) and a daily survey over 14 days which focused on day-to-day varying aspects. For this manuscript, we focus on the trait characteristics assessed once per wave. The study protocol was approved by the local ethics committee. Eligible for the study were all German-speaking adults who have been in a romantic relationship for at least one month. A further prerequisite for participation was that both partners of a couple were willing to take part in the study. After providing informed consent, participants completed online versions of the questionnaires. As remuneration for participation, participants received shopping vouchers worth 20 CHF/EUR as well as personalized feedback upon request for each wave if they provided sufficient data.

### Participants

Since this is an analysis of existing data, we did not conduct any a-priori power analysis. We conducted post-hoc sensitivity analyses for the research questions (RQs 1–3). Simulations suggested that the minimal detectable effect sizes with a power of .80 were *b* = .07 for actor and partner effects of relationship beliefs and their interactions with relationship duration on relationship satisfaction, *b* = .03 for associations between relationship beliefs and the trajectory of relationship satisfaction across time, and *b* = .09 for effects including the interaction of actor and partner effects of relationship beliefs.

An initial sample of 2315 individuals in a partnership (*n* = 1217 couples) participated in the study. We excluded 507 participants, as they or their partner a) did not provide information on relationship beliefs (*n* = 257 participants), b) started a partnership with a different partner during the study (*n* = 78 participants), c) did not provide information on relationship duration or age (*n* = 20 participants), d) did not agree with their partner regarding their relationship duration (i.e., a deviation >20% of reported relationship duration between partners; *n* = 56 participants), or (e) did not provide information on relationship satisfaction at any time point (*n* = 96 participants). Thus, the final study sample included *N* = 1808 participants in 904 couples.

At T1, women (*n* = 921) were aged 18–78 years (*Md* = 27.00, *SD* = 13.51), and men (*n* = 887) were aged 18–81 years (*Md* = 29.00, *SD* = 14.08). Couples have been in a relationship at T1 since *Md* = 4.90 years (*SD* = 10.49) with a range of 0.17–52.88 years. Regarding people’s marital status, most participants were never married (59.5%), while 35.0% were married, in a registered partnership (1.5%), separated (0.2%), divorced (3.2%), or widowed (0.6%).^3^ About a third of the sample (29.7%) had children (own children, or children of the partner living in the same household). Most participants (71.7%) lived with their partner (with or without children), 9.6% lived with their parents (and siblings), 8.9% lived alone, 8.4% lived in shared accommodation, 0.6% lived with their children (but not with their partner), and 0.9% reported different living arrangements. Participants reported residing in Germany (58.6%), Switzerland (30.0%), Austria (11.1%), or other countries (0.3%). We included data from both mixed-gender (96.6% of the couples) couples and same-gender couples (3.5% of the couples; 0.8% men–men and 2.7% women–women couples) in our analyses.

### Instruments

#### Main variables

##### Relationship beliefs

The Implicit Theories of Relationships Scale (ITR; Knee, 1998; German version by Schneewind et al., 2004) assesses two dimensions of relationship beliefs, namely, destiny and growth beliefs. Each subscale comprises four items, example items are “A successful relationship is mostly a matter of finding a compatible partner right from the start” (destiny beliefs) and “A successful relationship is mostly a matter of learning to resolve conflicts with a partner” (growth beliefs). Items are rated on a 5-point Likert scale, ranging from 1 (*do not agree at all*) to 5 (*agree completely*). We analyzed data on relationship beliefs from the first wave and the second wave, since it was not assessed at waves 3 and 4. Internal consistencies (McDonald’s omega) were ω = .72 (T1) and .78 (T2) for destiny beliefs and ω = .67 (T1) and .70 (T2) for growth beliefs and considered acceptable for the purpose of this study. Test–retest reliabilities across 5 months were *r*_tt_ = .65 (destiny beliefs) and *r*_tt_ = .59 (growth beliefs).

##### Relationship satisfaction

Relationship satisfaction was measured by the German version of the Relationship Assessment Scale (RAS; Hendrick, 1988; German version by Sander & Böcker, 1993). The scale comprises seven items, measuring how satisfied participants are with their current relationship (e.g., “In general, how satisfied are you with your relationship?”). Items are rated on a 5-point Likert scale and the description of the response options varies among the items. We analyzed data on relationship satisfaction from all four waves. Internal consistencies at the different time points were ω = .88 (T1), .89 (T2), .90 (T3), and .91 (T4). Test–retest reliabilities ranged from *r*_tt_ = .78 to .82 across 5 months (see Table S1).

##### Subjective change trajectories

Participants were asked to indicate subjective change trajectories in relationship satisfaction based on graphic illustrations regarding past relationship satisfaction and expectations for participants’ future relationship satisfaction. The two items were adapted from the German Socio-Economic Panel (GSOEP; DIW Berlin/SOEP, 2016). Participants were provided nine images (see Online Supplemental Table S2) to choose from and asked: “Which of the nine pictures best describes your relationship satisfaction from the beginning of the relationship until today?” (past), and “Which of the nine pictures best describes how you see your relationship developing in the future?” (future), respectively. We analyzed data on subjective change trajectories from the fourth wave since they were not assessed in earlier waves. Further, we focused on those four trajectories that were selected by at least 10% of the sample, namely, a) those reported no changes (flat line), b) those who reported a steady linear increase, c) those who reported ups and downs with an overall upward trend, and d) those who reported an increase between phases of stability (see Table 2).

#### Variables used for robustness check

##### Personality

The Big Five Inventory (BFI; John & Srivastava, 1999; German version by Rammstedt & Danner, 2016) assesses the five dimensions of the Five-Factor-Model (Big Five) of personality: neuroticism (N), extraversion (E), openness (O), conscientiousness (C), and agreeableness (A). The scale is widely used in personality research. Each subscale is measured by eight to ten items, summing up to a total number of 45 items. Example items are “I worry a lot” (N) or “I am talkative” (E). Participants are asked to describe themselves by rating their agreement with the statements on a 5-point Likert scale, ranging from 1 (*strongly disagree*) to 5 (*strongly agree*). We analyzed data of the Big Five dimensions from T1. Internal consistencies were ω = .82 (N), .90 (E), .91 (O), .85 (C), and .82 (A).

##### Self-esteem

The Rosenberg Self-Esteem Scale (RSE; Rosenberg, 1965; German version by von Collani & Herzberg, 2003) comprises 10 items, measuring how valuable participants perceive themselves (e.g., “I feel that I have a number of good qualities.”). Items are rated on a 4-point Likert scale ranging from 1 (*strongly agree*) to 4 (*strongly disagree*). We analyzed data of self-esteem from T1. Internal consistency was ω = .94.

##### Life satisfaction

The Satisfaction with Life Scale (SWLS; Diener et al., 1985; German version by Glaesmer et al., 2011) comprises 5 items, measuring how happy people are with their lives (e.g., “In most ways my life is close to my ideal.”). Items are rated on a 7-point Likert scale ranging from 1 (*strongly disagree*) to 7 (*strongly agree*). We analyzed data of life satisfaction from T1. Internal consistency was ω = .84.

##### Attachment orientations

The Experiences in Close Relationships-Relationship Questionnaire (ECR; Fraley et al., 2011; used in a German version) assesses attachment security on two dimensions: attachment anxiety and avoidance. The questionnaire measures how comfortable a person is with being close to a partner. The subscale avoidance comprises 6 items (e.g., “I don’t feel comfortable opening up to this person.”) and the subscale anxiety has 3 items (e.g., “I’m afraid that this person may abandon me”). Items are rated on a 7-point Likert scale ranging from 1 (*strongly disagree*) to 7 (*strongly agree*). We analyzed data of attachment security from T1. Internal consistencies were ω = .78 (avoidance) and ω = .75 (anxiety).

### Data analysis

**Figure 1.**
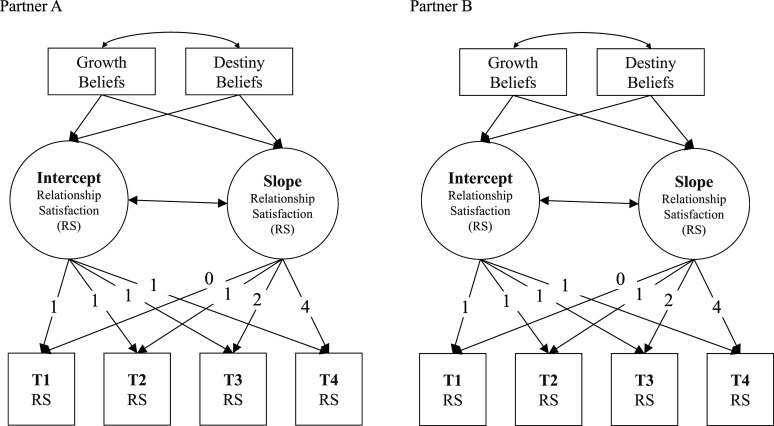
Dyadic Growth Curve Model. *Note.* Schematic representation of the dyadic growth curve models for predicting relationship satisfaction. In order to reduce complexity, the partner effects and covariances between partners are not shown. Numbers represent the loadings of the indicators for the latent intercept (=1; all time points were equally weighted for the intercept) and the slope (=0–4, representing the length of the intervals among the time points; the interval between times 3 and 4 was twice as long as the intervals between times 1, 2, and 3), respectively.

Scale reliabilities were estimated using McDonald’s omega for categorical data (Flora, 2020). For examining research questions 1 to 3, data were analyzed using longitudinal multilevel models (i.e., growth curve models). Because we also aimed at considering partner effects, we used dyadic growth curve models (e.g., Iida et al., 2023; Kashy & Donnellan, 2008; Kenny et al., 2006). We modeled the trajectory of relationship satisfaction across the four measurement time points for each dyad, regressing the trajectories of relationship satisfaction on the predictors assessed at baseline (see Figure 1). This allowed us to examine the associations between relationship beliefs and one’s own level and change (i.e., actor effects) as well as the partner’s level and change (i.e., partner effects) of relationship satisfaction.

In line with earlier research on romantic dyads (e.g., Jackson et al., 2014), we examined whether gender moderated the effects and we controlled for age and relationship duration.^4^ As a robustness check (RQ5), we computed our main analyses additionally with the inclusion of the Big Five dimensions, self-esteem, satisfaction with life, and attachment orientations. All psychological variables (i.e., relationship beliefs, relationship satisfaction, and Big Five dimensions) were *z*-standardized (using the full sample), and all variables with a meaningful metric (i.e., age and relationship duration) were grand-mean centered. For both age and relationship duration, the metric was additionally adjusted to 10-year intervals (i.e., relationship duration in years was divided by 10) to prevent the resulting regression weights from becoming too small and to prevent the variance to be too large compared to the other variables. Relationship duration was averaged across both partners since there was a very high agreement on average (ICC[1,1] = .995). Gender was effect coded (−1 = women, 1 = men).

For examining research question 4, we focused on those four trajectories of past and future relationship satisfaction that were selected by at least 10% of the sample, since some patterns were chosen very rarely (e.g., a steady decline in past relationship satisfaction was only reported by 10 people). We predicted these four trajectories by age, gender, relationship duration, and relationship beliefs, and the interaction between gender and relationship beliefs, while also allowing a random intercept for couples. For this purpose, we conducted Bayesian multinomial regression analyses since this also allows for considering random intercepts. We used uninformative priors and ran the model with 4 chains using 3000 iterations each, which resulted in good model convergence (all scale reduction factors *R* < 1.05). For these analyses, we report and interpret 95% credible intervals for the parameter estimates.

For research question 6, we ran the same dyadic growth curve models as for research questions 1–3, predicting the levels and trajectories of relationship beliefs by baseline levels of relationship satisfaction. Since relationship beliefs were only assessed in waves 1 and 2, these models only covered the data assessed in the first two waves.

For all our analyses, we used R (Version 4.3.0; R Core Team, 2023) and the R-packages *brms* (Version 2.19.0; Bürkner, 2021), *doParallel* (Version 1.0.17; Weston & Calway, 2022), *foreach* (Version 1.5.2; Weston & Calway, 2022), *lavaan* (Version 0.6.9; Rosseel, 2012), *nlme* (Version 3.1.152; Pinheiro & Bates, 2000; R Core Team, 2021), *papaja* (Version 0.1.0.9999; Aust & Barth, 2020), *rsample* (Version 1.2.0; Frick et al., 2023), *psych* (Version 2.1.9; Revelle, 2021), *sjPlot* (Version 2.8.9; Lüdecke, 2021), and *tidyverse* (Version 2.0.0; Wickham et al., 2019).

## Results

### Preliminary analyses

Means and standard deviations for all study variables are presented in Table S1, and zero-order correlations are shown in Table S1. On average, participants reported high levels of growth beliefs (*M* = 4.11, *SD* = 0.62, on a scale ranging from 1 to 5) and comparably lower levels of destiny beliefs (*M* = 2.88, *SD* = 0.83, on a scale ranging from 1 to 5) which is in line with earlier studies (e.g., Knee et al., 2001). Furthermore, partners’ beliefs were somewhat correlated for growth beliefs (*r* = .23, *p* < .001) and destiny beliefs (*r* = .22, *p* < .001).

Due to the attrition during the course of the study, we compared the baseline characteristics of those participants who completed all waves (“completers”; 48.3%) with those who missed one or more waves (“dropouts”; 51.7%). Analyses showed that the completers did not differ from the dropouts in terms of age (*r* = .03, *p* = .201), relationship duration (*r <* .01, *p* > .811), or destiny (*r* = −.04, *p* = .148) and growth beliefs (*r* = −.03, *p* = .130). However, dropouts tended to be more often men (*r* = .05, *p* = .028) and less satisfied with their relationship at the beginning of the study (*r* = −.09, *p* < .001).

Further, we examined whether relationship satisfaction was invariant across the four time points, which was confirmed: The models did not suggest noteworthy deterioration in model fit under both metric and scalar invariance (see Table S3). However, to reduce model complexity, we used manifest scores of all variables for subsequent analyses.

### Predicting relationship satisfaction trajectories by relationship beliefs (RQs 1–3)

For examining research questions 1 to 3, we started with a basic model predicting relationship satisfaction by time, while allowing for random intercepts for couples and random slopes for time. Comparing linear effects of time with quadratic effects of time did not improve model fit (BIC_ModelLinear_ = 11,740 vs. BIC_ModelQuadratic_ = 11,745, Δχ^2^[Δ*df* = 1] = 3.54, *p* = .06), and we therefore only considered linear trajectories. We compared this model with a model that additionally allows different fixed and random effects across time for men and women. A comparison of those two models suggested a better fit of the latter model that distinguished between genders (BIC_ModelConstrained_ = 11,740 vs. BIC_ModelUnconstrained_ = 10,770, Δχ^2^[Δ*df* = 10] = 1056.00, *p* < .001). We additionally examined whether the trajectories differ between same-gender and mixed-gender couples by including the interaction between actor and partner gender, which suggested no differences in the main effects or the trajectories across time in relationship satisfaction. We therefore included both same-gender and mixed-gender couples in the analyses (cf. Weidmann & Chopik, 2022). In subsequent analyses, we only included actor gender but not partner gender due to their strong association (*r* = −.93) and for avoiding multicollinearity issues.

For the main analyses, we predicted relationship satisfaction by time, age, gender, relationship duration, actor and partner effects of relationship beliefs, and the interaction between actor and partner effects. We also included the two- and three-way interactions between time, gender, and relationship beliefs, as well as the interactions of relationship duration, gender, and relationship beliefs.^5^ Results are presented in Table 1.

**Table 1.** Fixed effects for growth and destiny beliefs, time, and relationship duration, and their interactions on relationship satisfaction.

	*b*	95% CI	*t*	*df*	*p*
Main Effects					
Intercept	−.08	[−0.14, −0.02]	−2.59	4254	.010
Age	.02	[−0.04, 0.07]	0.56	4254	.575
Gender	**−.05**	[−0.08, −0.01]	−2.82	4254	.005
Duration	**−.12**	[−0.21, −0.04]	−3.00	900	.003
Time	**−.11**	[−0.14, −0.08]	−7.18	4254	<.001
Growth Beliefs (actor effect)	.02	[−0.03, 0.06]	0.85	4254	.396
Destiny Beliefs (actor effect)	**.06**	[0.01, 0.10]	2.59	4254	.010
Growth Beliefs (partner effect)	−.02	[−0.07, 0.02]	−1.07	4254	.283
Destiny Beliefs (partner effect)	.03	[−0.01, 0.07]	1.31	4254	.190
Growth Beliefs (actor × partner effect)	**.08**	[0.02, 0.14]	2.78	900	.006
Destiny Beliefs (actor × partner effect)	−.01	[−0.06, 0.05]	−0.22	900	.826
Interactions With Time					
Time × Growth Beliefs (actor effect)	**.03**	[0.00, 0.05]	2.35	4254	.019
Time × Destiny Beliefs (actor effect)	.00	[−0.02, 0.03]	0.17	4254	.862
Time × Growth Beliefs (partner effect)	.00	[−0.03, 0.02]	−0.37	4254	.712
Time × Destiny Beliefs (partner effect)	−.02	[−0.04, 0.00]	−1.56	4254	.119
Time × Growth Beliefs (actor × partner effect)	.02	[−0.01, 0.04]	1.17	4254	.243
Time × Destiny Beliefs (actor × partner effect)	**−.03**	[−0.06, 0.00]	−2.17	4254	.030
Interactions With Relationship Duration					
Duration × Growth Beliefs (actor effect)	.02	[−0.02, 0.06]	0.98	4254	.328
Duration × Destiny Beliefs (actor effect)	.03	[−0.01, 0.07]	1.42	4254	.155
Duration × Growth Beliefs (partner effect)	.02	[−0.02, 0.06]	0.95	4254	.341
Duration × Destiny Beliefs (partner effect)	.01	[−0.03, 0.05]	0.41	4254	.679
Duration × Growth Beliefs (actor × partner effect)	−.01	[−0.07, 0.04]	−0.53	4254	.596
Duration × Destiny Beliefs (actor × partner effect)	.03	[−0.02, 0.08]	1.30	4254	.193

*Note.* Random effects and interactions with gender are not shown; see Online Supplemental Table S4 for full results. Age = Age in decades (grand-mean centered), Duration = Relationship Duration in decades (grand-mean centered), Time = Time in years. Gender: −1 = women, 1 = men. All other variables are *z*-standardized. Estimates in bold are significant (*p* < .05).

**Figure 2.**
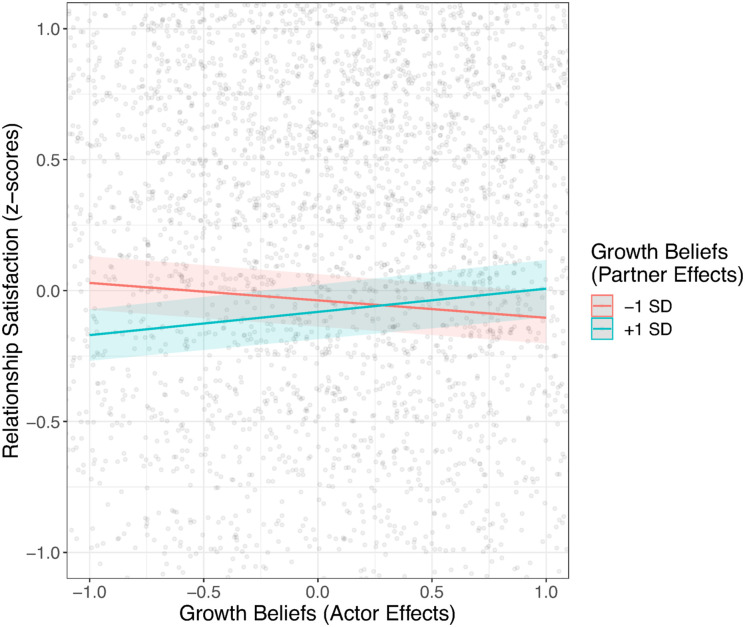
Actor–Partner Interactions of Growth Beliefs on Relationship Satisfaction Levels. *Note.* Gray dots show the raw data, and the colored lines show the interaction effects.

Table 1 shows that, for the average couple, relationship satisfaction declined over time (i.e., about 11% of a standard deviation per year), and with longer relationship duration (i.e., about 12% of a standard deviation per 10-year interval). Relationship satisfaction differed by gender, suggesting that women reported higher relationship satisfaction than men. Regarding relationship beliefs, those with stronger destiny beliefs reported higher levels of relationship satisfaction. For growth beliefs, there were no main effects but interactions between actor and partner effects: Highest relationship satisfaction levels were reported in those couples where both partners reported high or low levels of growth beliefs, while relationship satisfaction was lower when partners disagreed in their growth beliefs (see Figure 2).

**Figure 3.**
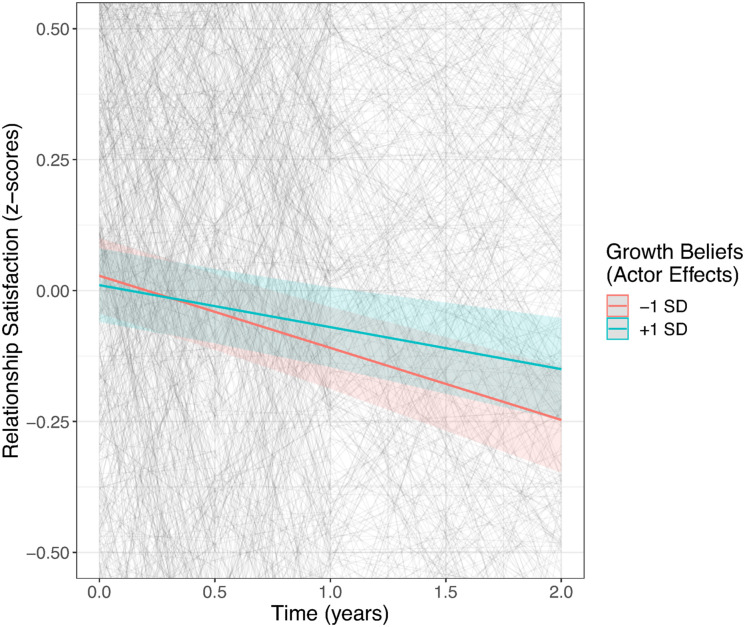
Trajectories of Relationship Satisfaction Across Time Depending on Growth Beliefs. *Note.* Gray lines show the raw data, and the colored lines show the fitted linear trajectories.

**Figure 4.**
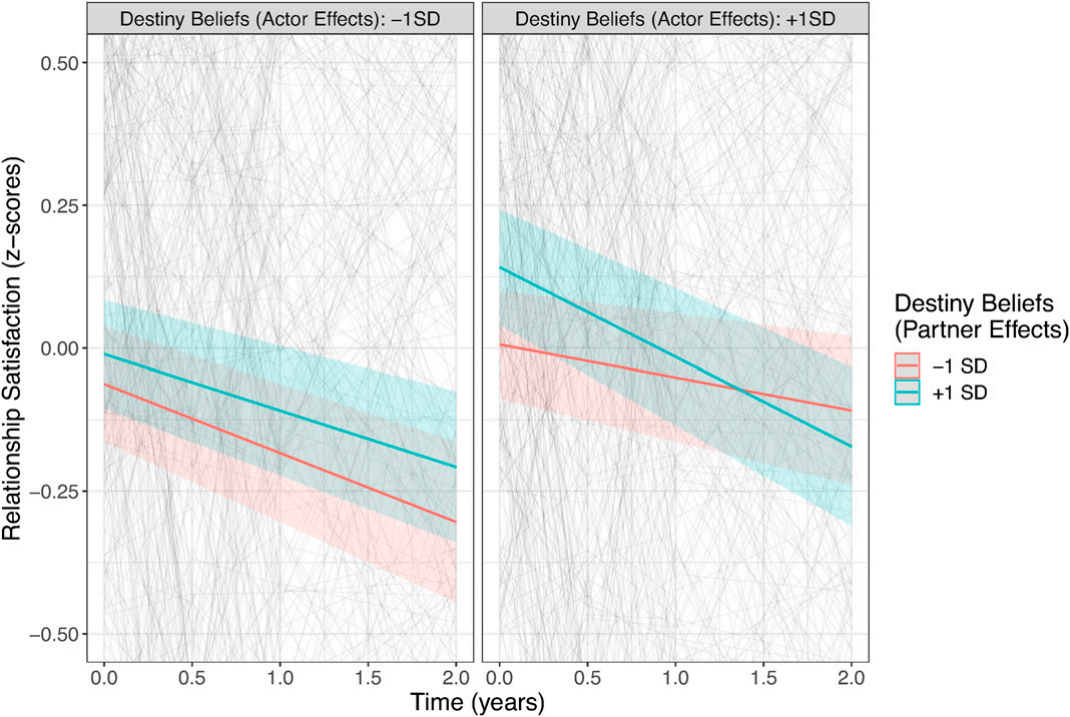
Trajectories of Relationship Satisfaction Across Time Depending on Actor–Partner Interactions in Destiny Beliefs. *Note.* Gray lines show the raw data, and the colored lines show the fitted linear trajectories.

Regarding the effects over time, growth beliefs predicted change in relationship satisfaction across time: Those with stronger growth beliefs reported less decline in their relationship satisfaction over the course of the study (see Figure 3). Even though individuals with stronger growth beliefs (i.e., one standard deviation above the mean) still experienced a significant decline, the rate of decline was nearly halved in comparison to individuals with lower growth beliefs (i.e., one standard deviation below the mean). Further, the trajectory of relationship satisfaction over time was also predicted by the interaction between actor and partner effects of destiny beliefs, suggesting the strongest declines for those couples where both partners report high destiny beliefs (see Figure 4). Finally, the association between relationship duration and relationship trajectories was unrelated to relationship beliefs.

While there were several additional significant results that were only present in one gender (see Online Supplemental Table S4), these effects disappeared when including additional individual difference variables (see below) and were therefore not further examined in detail.

#### Robustness check (RQ4)

We additionally included the Big Five dimensions, self-esteem, satisfaction with life, and attachment orientations to check whether the previously reported findings regarding relationship beliefs could also be explained by other individual difference constructs (see Table S5): Relationship satisfaction levels were also positively predicted by agreeableness and life satisfaction, and negatively predicted by attachment anxiety and avoidance. Regarding longitudinal effects, those with high levels of extraversion and low levels of self-esteem at baseline reported weaker declines in relationship satisfaction over time. At the same time, results for relationship beliefs mostly remained unchanged: There was still a main effect for destiny beliefs and an interaction between actor and partner effects of growth beliefs on relationship satisfaction levels, while growth beliefs still predicted the slope of relationship satisfaction across time.

### Associations between growth and destiny beliefs and subjective trajectories of past and future relationship satisfaction (RQ5)

To examine research question 5, we predicted the different subjective trajectories (dummy coded) of past and future relationship satisfaction by growth and destiny beliefs, while controlling for age, gender, and relationship duration, and allowing a random intercept for couples. Results are presented in Table 2.

**Table 2.** Multinomial regression results for associations for predicting subjective trajectories of past and future relationship satisfaction by growth and destiny beliefs.

	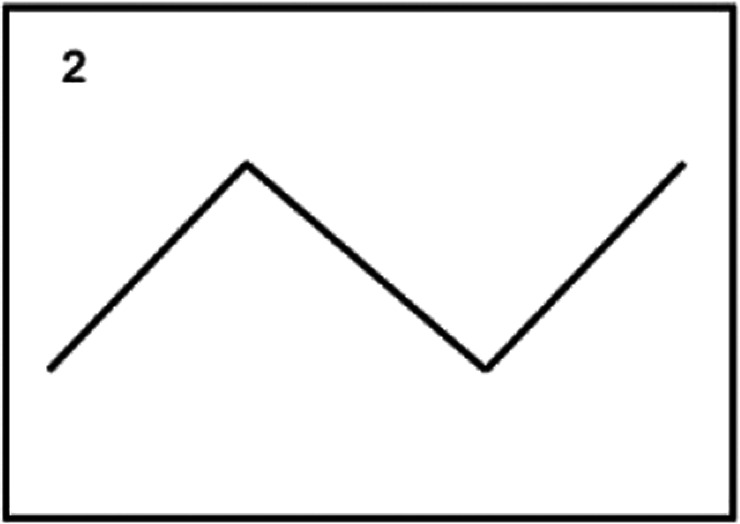		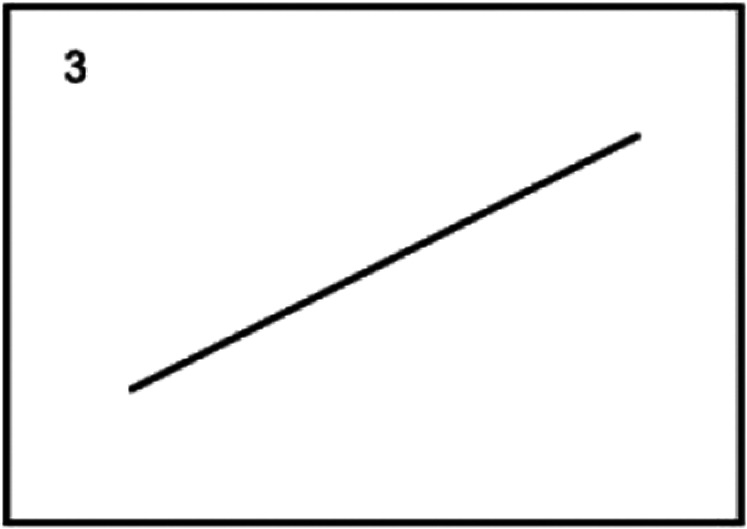		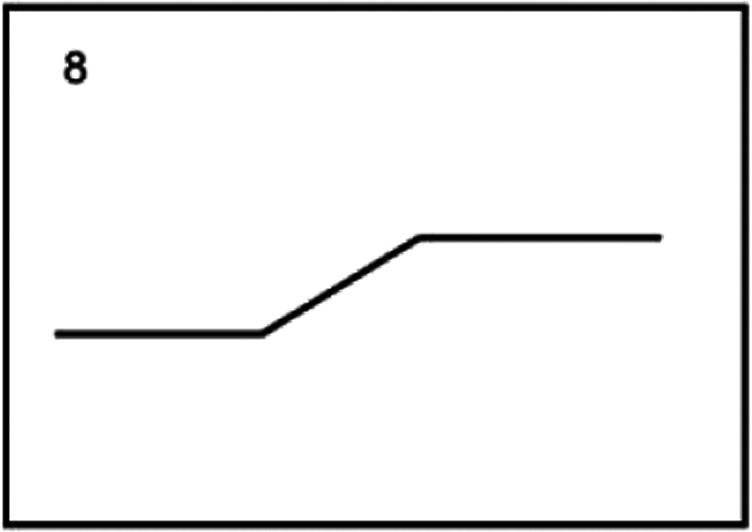	
	*b*	95% *CI*	*b*	95% *CI*	*b*	95% *CI*
Past relationship satisfaction trajectory						
Intercept	0.23	[−0.28, 0.64]	**0.94**	[**0.63, 1.22]**	**0.55**	[**0.20, 0.87]**
Gender	0.19	[−0.11, 0.49]	0.22	[−0.03, 0.48]	0.11	[−0.16, 0.39]
Age	−0.05	[−0.10, 0.00]	0.01	[−0.02, 0.04]	0.01	[−0.03, 0.04]
Duration	3.81	[−1.94, 9.68]	−2.23	[−6.39, 1.93]	−0.28	[−4.71, 3.99]
Growth Beliefs	0.30	[−0.03, 0.61]	**0.31**	[**0.06, 0.57]**	0.14	[−0.13, 0.41]
Destiny Beliefs	0.02	[−0.29, 0.35]	0.08	[-0.20, 0.34]	0.11	[−0.18, 0.40]
Gender × Growth Beliefs	−0.02	[-0.33, 0.30]	0.15	[−0.10, 0.42]	**0.29**	[**0.03, 0.56]**
Gender × Destiny Beliefs	0.07	[−0.25, 0.37]	**0.38**	[**0.13, 0.66]**	0.12	[−0.15, 0.39]
Future relationship satisfaction trajectory						
Intercept	−0.33	[−0.99, 0.10]	**1.05**	[**0.77, 1.32]**	**1.02**	[**0.78, 1.27]**
Gender	0.14	[−0.14, 0.44]	0.02	[−0.20, 0.26]	−0.16	[−0.38, 0.07]
Age	−0.03	[−0.07, 0.01]	−0.02	[−0.05, 0.01]	−0.03	[−0.05, 0.00]
Duration	−2.27	[−7.15, 2.75]	−2.42	[−6.11, 1.21]	−1.03	[−4.32, 2.29]
Growth Beliefs	**0.39**	[**0.08, 0.70]**	**0.32**	[**0.07, 0.57]**	0.23	[0.00, 0.46]
Destiny Beliefs	0.04	[−0.26, 0.33]	0.24	[0.00, 0.49]	0.02	[-0.20, 0.25]
Gender × Growth Beliefs	**−0.34**	[−**0.66,** −**0.04]**	−0.10	[−0.34, 0.14]	−0.12	[−0.34, 0.11]
Gender × Destiny Beliefs	−0.10	[−0.41, 0.19]	0.04	[−0.19, 0.28]	0.00	[−0.22, 0.22]

*Note.* Comparison trajectory = No change (flat line). Age in decades (grand-mean centered), Duration = Relationship duration in decades (grand-mean centered), Gender: −1 = women, 1 = men. Estimates in bold are significant (95% CI does not contain zero).

Table 2 shows that participants with higher growth belief scores were more likely to report a steady increase in their relationship satisfaction over time compared to those who reported no change in their relationship satisfaction. Two effects were qualified by gender: In men (but not women), destiny beliefs also went along with a higher likelihood of reporting a steady increase over time. Further, women with low growth beliefs were more likely to report the last pattern (i.e., an increase between phases of stability, see Table 2 for patterns).

Regarding future relationship satisfaction, those with higher growth belief scores were more likely to expect a steady increase or ups and downs with an overall upward trend in their relationship satisfaction in the future, compared to those who expected no change. Additionally, women with high growth beliefs were more likely to select the first pattern (ups and downs with an overall upward trend).

### Predicting relationship belief trajectories by relationship satisfaction (RQ6)

For examining whether the effects go in both directions, and whether baseline levels of relationship satisfaction also predict the trajectories of relationship beliefs, we repeated the analyses of RQs 1-3 and predicted relationship beliefs by time, age, gender, relationship duration, actor and partner effects of relationship satisfaction, and the interaction between actor and partner effects. We also included the two- and three-way interactions between time, gender, and relationship beliefs, as well as the interactions of relationship duration, gender, and relationship satisfaction. Results are presented in Table 3.

**Table 3.** Fixed effects for relationship satisfaction, time, and relationship duration, and their interactions on growth and destiny beliefs.

	*b*	95% CI	*T*	*df*	*p*
**Growth Beliefs**					
*Main Effects*					
Intercept	.04	[−0.01, 0.10]	1.62	2192	.106
Age	**−.21**	[−0.26, −0.15]	−7.47	2192	<.001
Gender	−.03	[−0.08, 0.01]	−1.53	2192	.127
Duration	**.16**	[0.08, 0.24]	4.16	818	<.001
Time	**.16**	[0.10, 0.21]	5.79	2192	<.001
Satisfaction (actor effect)	.03	[−0.02, 0.08]	1.06	2192	.289
Satisfaction (partner effect)	−.05	[−0.10, 0.00]	−1.92	2192	.055
Satisfaction (actor × partner effect)	−.04	[−0.09, 0.00]	−1.86	818	.063
*Interactions With Time*					
Time × Satisfaction (actor effect)	**.07**	[0.01, 0.13]	2.28	2192	.023
Time × Satisfaction (partner effect)	.05	[−0.01, 0.11]	1.64	2192	.101
Time × Satisfaction (actor × partner effect)	−.02	[−0.06, 0.03]	−0.67	2192	.501
*Interactions With Relationship Duration*					
Duration × Satisfaction (actor effect)	.02	[−0.03, 0.07]	0.68	2192	.498
Duration × Satisfaction (partner effect)	.02	[−0.03, 0.07]	0.75	2192	.453
Duration × Satisfaction (actor × partner effect)	.02	[−0.01, 0.06]	1.33	2192	.185
**Destiny Beliefs**					
*Main Effects*					
Intercept	−.03	[−0.09, 0.02]	−1.08	2192	.278
Age	**.15**	[0.10, 0.21]	5.41	2192	<.001
Gender	.02	[−0.02, 0.07]	1.04	2192	.299
Duration	**−.12**	[−0.19, −0.04]	−2.94	818	.003
Time	**−.09**	[−0.14, −0.04]	−3.72	2192	<.001
Satisfaction (actor effect)	**.06**	[0.01, 0.11]	2.38	2192	.017
Satisfaction (partner effect)	**.06**	[0.01, 0.12]	2.46	2192	.014
Satisfaction (actor × partner effect)	.04	[−0.01, 0.09]	1.68	818	.093
*Interactions With Time*					
Time × Satisfaction (actor effect)	−.03	[−0.08, 0.03]	−0.97	2192	.332
Time × Satisfaction (partner effect)	.01	[−0.05, 0.06]	0.22	2192	.825
Time × Satisfaction (actor × partner effect)	.00	[−0.04, 0.04]	−0.01	2192	.989
*Interactions With Relationship Duration*					
Duration × Satisfaction (actor effect)	.04	[−0.01, 0.10]	1.67	2192	.095
Duration × Satisfaction (partner effect)	.01	[−0.04, 0.06]	0.41	2192	.683
Duration × Satisfaction (actor × partner effect)	.01	[−0.02, 0.05]	0.57	2192	.567

*Note.* Random effects and interactions with gender are not shown; see Online Supplemental Table S4 for full results. Age = Age in decades (grand-mean centered), Duration = Relationship Duration in decades (grand-mean centered), Time = Time in years. Gender: −1 = women, 1 = men. All other variables are *z*-standardized. Satisfaction = Relationship Satisfaction. Estimates in bold are significant (*p* < .05).

Table 3 shows that growth belief levels were higher among younger couples and couples who have been together for a longer time. Overall, growth beliefs increased from wave 1 to wave 2. While growth belief levels were independent of relationship satisfaction, those who reported higher relationship satisfaction at baseline reported stronger increases in growth beliefs over time.

Destiny beliefs were associated with older age and shorter relationship durations. In contrast to growth beliefs, they declined over time. Furthermore, higher baseline relationship satisfaction was associated with stronger destiny beliefs in oneself and one’s partner. However, relationship satisfaction did not predict changes in destiny beliefs over time. Again, there were some effects of relationship satisfaction on relationship beliefs that were only present in one gender (see in Online Supplemental Tables S6 and S7).

## Discussion

The present study examined the role of relationship beliefs (i.e., growth and destiny beliefs) for relationship satisfaction. Results indicate that destiny beliefs were associated with a higher level of relationship satisfaction, and higher growth beliefs were associated with a less steep decline in relationship satisfaction over time. At the same time, higher relationship satisfaction also went along with an increase in growth beliefs over time, suggesting that relationship beliefs and relationship satisfaction are intertwined.

Furthermore, the study also suggested that those participants who endorsed more growth beliefs tended to describe the past trajectory of their relationship satisfaction as more positive than those with lower growth beliefs. Therefore, the post-hoc subjective perception of the trajectories corroborates the findings on the measured longitudinal trajectories for relationships satisfaction. Further, those with higher growth beliefs also held more often an optimistic view on the future development of their relationship. This seems to describe the core idea of growth beliefs well, namely, the belief that relationships grow and can get better over time (e.g., Knee, 1998). This beneficial outlook toward the future might be relevant for the maintenance of relationships: Those participants who believe that things can turn for the better are presumably more likely to invest in their relationships (e.g., making plans together, acquiring shared possessions), which have been found to predict commitment and to prevent breakup (e.g., Goodfriend & Agnew, 2008), or might also be more inclined to seek couples’ therapy to work on the relationship. Earlier work also demonstrated that optimism in close relationships is associated with higher relationship satisfaction due to a higher level of perceived support (Srivastava et al., 2006).

However, these mechanisms have not been assessed in the present study and it is up to future studies to investigate whether this is indeed the case. Interestingly, while the large majority of couples (i.e., 84%) reported a decline in their relationship satisfaction over the course of the study, most participants (i.e., 66% of men and 72% of women) reported a subjective trajectory with an overall upward trend (with or without ups and downs) in their past relationship satisfaction. This finding was even more pronounced regarding future relationship satisfaction: About 81% of participants expected a positive development of their relationship satisfaction in the future. These findings suggest that participants’ subjective trajectories of relationship satisfaction might be prone to optimistic bias, as has been reported earlier for subjective trajectories (e.g., Gander & Wagner, 2022).

In line with previous research, we found a decrease in relationship satisfaction (e.g., Bühler et al., 2021b) as well as gender differences in relationship satisfaction (see Butzer & Campbell, 2008). Also, in line with previous studies (e.g., Joel et al., 2020), the present study supported that partner effects were less relevant than actor effects: Partner effects were mostly smaller in size than actor effects, and only one partner effect reached significance (i.e., the three-way interaction between destiny beliefs, gender, and relationship duration). McNulty and colleagues (2021) note in their paper that partner effects do exist, but they are usually detectable in moderation analyses which is in line with our findings. Thus, considering the perspective of the partner added little to understanding levels of and changes in relationship satisfaction. On the other hand, we did not consider different configurations of destiny and growth beliefs (e.g., having simultaneously high growth and low destiny beliefs), which have been argued to be relevant in previous studies (e.g., Knee et al., 2001).

The relationship beliefs between partners showed small correlations, suggesting that while partners may share some similar beliefs, there are also individual differences in how they perceive and approach the relationship. At the same time, the interaction between their beliefs revealed to be relevant for both levels (growth beliefs) and trajectories (destiny beliefs) of relationship satisfaction: Highest relationship satisfaction levels were reported if both partners reported high or low growth beliefs, while couples where both partners reported high destiny beliefs showed the steepest decline in relationship satisfaction over time. At the same time, these couples reported higher relationship satisfaction at the beginning of the study and reported similar levels of relationship satisfaction to those with lower levels of destiny beliefs at the end of the 2-year period. Thus, it is possible that the initial beneficial effect of (shared) high destiny beliefs might erode over time. Nonetheless, these results suggest that the configurations of relationship beliefs can also play a relevant role and further research on their effects in relationships is warranted.

As a robustness check, we have also examined whether our findings could be explained by differences in several individual difference variables, namely, the Big Five dimensions, self-esteem, satisfaction with life, and attachment orientations, which was not the case. Thus, the predictive validity of relationship beliefs seems independent of these individual differences and can explain variance in levels and changes of relationship satisfaction above and beyond some of the most potent predictors of relationship outcomes (e.g., Joel et al., 2020; Soto, 2019).

Noteworthy, all effects in the present study were small, especially the moderators of satisfaction trajectories over time. While this is not surprising, given the failure to identify relevant moderators of trajectories in past studies (e.g., Joel et al., 2020), it still suggests relationship beliefs do not play a major role in relationship satisfaction overall. This makes sense when considering that relationship satisfaction and stability is a complex interaction between individual, relationship, and external factors (Le et al., 2010). However, we only examined a time frame of 2 years. Over a longer period of time, the comparably small effects might be of greater importance. Thus, relationship beliefs might be especially relevant for long-term outcomes given their assumed amenability to change.

This raises the question of whether these beliefs are stable or whether and how they can be deliberately altered. At the same time, the test–retest reliabilities for relationship beliefs over 5 months were relatively low, especially when compared to more stable constructs like Big Five personality traits (e.g., *r*_tt_ ≥ .77 across a period of 5 months; Bühler et al., 2021b), but also lower than relationship satisfaction (e.g., *r*_tt_ ≥ .78 in the present study). These stability discrepancies might highlight two points to consider for the interpretation of the present findings: First, the low stability might add to the explanation of the relatively low associations of relationship beliefs with Big Five dimensions—relationship beliefs might be influenced by a broader range of contextual and situational factors that may fluctuate more than core personality traits. Second, this might suggest that these beliefs can potentially change over relatively short periods (i.e., 5 months) and could therefore be indicative of a higher malleability of relationship beliefs, which could have important implications for interventions targeting relationship beliefs. Indeed, the present study found increases in growth beliefs over time for those with a high initial relationship satisfaction.

Although to the best of our knowledge, no intervention based on relationship beliefs has been developed, there is some evidence regarding the effectiveness of interventions regarding other beliefs related to growth mindsets (Yeager & Dweck, 2020), and several experimental studies successfully manipulated relationship beliefs, at least in the short term (e.g., Chen et al., 2012; Franiuk et al., 2004; Thompson et al., 2020). Therefore, we argue that relationship beliefs warrant further scrutiny as a potential intervention to improve relationship quality. Overall, the findings of this study indicate that couples’ lay beliefs about their relationship might affect the development and trajectory of relationship quality to a certain extent. While the observed effect sizes are small, it appears that stronger growth beliefs could be a factor in why some couples maintain higher relationship satisfaction over time.

### Limitations

Several limitations of the present study have to be considered. First and foremost, the primary goal of this study was to examine whether relationship beliefs predict levels and changes of relationship satisfaction. The lower test–retest reliabilities of relationship beliefs compared to relationship satisfaction raises the question of whether relationship satisfaction is a better predictor of relationship beliefs than vice versa. Although the prediction of growth belief trajectories by relationship satisfaction yielded a (numerically, but not significantly) higher effect than the prediction of relationship satisfaction trajectories by growth belief, data for relationship beliefs were only available for two waves. Therefore, the trajectories of relationship beliefs differed from those of relationship satisfaction (which covered four waves), which limits direct comparability.

Of course, we are not able to establish any causal connections between the studied variables or whether causal effects are due to third variables remains unknown. The present study is only able to show that relationship beliefs and relationship satisfaction are interconnected in the sense that one can predict the levels and trajectories of the other.

Further, we did not assess whether the current partner is perceived as the “ideal” partner which has been suggested to be an important moderator of the relationship between relationship beliefs and relationship outcomes (Franiuk et al., 2002, 2004). The effects of destiny beliefs might differ depending on whether one believes to have already found their ideal partner or not. Thus, it is possible that the effects of relationship beliefs were underestimated by not considering this variable. On the other hand, individuals may not be able to assess whether their partner is “ideal” as this evaluation may change over time. Especially in the early stages of a relationship, individuals may think they have found the ideal partner and then lose this image over time when, for instance, daily life reveals weaknesses and downsides of the partners’ personality. Therefore, being with the “ideal” partner may not be a reliable covariate to control for.

Additionally, the findings of the present study cannot be generalized to other beliefs among couples (e.g., sexual beliefs, Uppot et al., 2024) or general incremental beliefs which are associated with thinking your life will get better over time (e.g., growth theories, Busseri & Samani, 2019).

Furthermore, the present study covered a broad array of couples in various stages of the relationship (and life), including both rather new and long-term relationships. Although this offers the benefit of representing romantic relationships across the whole life span, it also comes with a limitation: Given that life stages and relationship satisfaction are linked to, for example, different developmental crises (e.g., Erikson, 1959), and roles (Roberts & Wood, 2006) such as parenting or retirement, the broad sample’s life experiences might blur the effects of relationship beliefs on relationship satisfaction. For example, it is possible that relationship beliefs play a larger role in specific stages of some of our participants’ lives or their relationships while these processes have already been completed or not yet started in other participants (e.g., to have children, marriage, shared accommodation, and retirement). While we did consider interactions of relationship beliefs with (linear) relationship duration and age, the moderating effects of age and relationship duration might follow a non-linear pattern, which would have biased the results. For subjective trajectories of relationship satisfaction, it has to be noted that the subjective trajectories did not ask about the development of relationship satisfaction since the start of the study but since the start of the relationship; thus, the subjective trajectories only partially overlapped with the measured trajectories.

Further, 51.7% of the sample missed at least one assessment and we therefore know nothing about their longitudinal trajectory of relationship satisfaction. Previous research by Park and colleagues (2021) indicated that participants participating with their partner (compared to without partner) are significantly less likely to breakup over time. Our attrition analyses suggested that those who dropped out were similar in most characteristics to those who completed all waves, with the exception for gender (men dropped out more often) and relationship satisfaction (less satisfied people dropped out more often). While these baseline differences were small by conventional standards (all *r* ≤ .10), it might still have biased our findings. We suspect that some of these couples who dropped out separated during the course of the study and therefore also ended their participation in the study. Thus, our results may only concern couples in a relationship and may not necessarily be generalizable to couples who separated. However, when looking at the (very limited) available data of couples who separated (*n* = 49), results showed that separating was widely unrelated to growth and destiny beliefs (*r* = −.01 for both), suggesting that it is unlikely that separating couples would have influenced the findings.

Some of the observed effects in our study were at or marginally below the minimal detectable effect size identified in sensitivity analyses. Given the complexity of our data, the sensitivity analyses only yield approximate estimates of this threshold. Therefore, while our study was adequately powered to also identify small effects, the proximity of some of our results to this lower detectability boundary warrants a cautious interpretation of their robustness.

Although having both partners’ perspectives is advantageous, this could also represent a limitation when both partners agree to participate: Since such couples have been found to report higher levels of relationship satisfaction than studies that did not require both partners to participate (Barton et al., 2020), our sample might be biased toward higher levels of satisfaction. Further, our study only covered a period of 2 years. While this is a longer period than previous studies of relationship beliefs have covered, it is a relatively short period of time in the realm of romantic relationships—especially given that in our sample, the median relationship duration at baseline was close to 5 years. This of course limits our conclusions of within-person effects of this time span. Further, previous research suggests that the trajectories of relationship satisfaction could follow more complex patterns (e.g., Bühler et al., 2021b). While we examined the possibility of curvilinear effects which did not result in a considerably improved description of the data, our dataset only consisted of four measurement waves. More waves across longer time intervals might be needed for a reliable assessment of more complex patterns.

Finally, while we attempted to assess the robustness of our findings and controlled for baseline levels of relevant predictors within the relationship domain, our dataset did not encompass additional pertinent predictors highlighted in the meta-analysis by Joel et al. (2020). Consequently, it remains possible that the observed effects of relationship beliefs might also be partly attributable to these other variables, which were not accounted for in our study.

## Conclusion

Relationship beliefs (i.e., growth and destiny beliefs) and relationship satisfaction are interconnected: The belief that one’s relationship is meant to be (i.e., destiny beliefs) goes along with higher current levels of life satisfaction. Further, the belief that a relationship can be cultivated (i.e., growth beliefs) predicts a less steep decline of relationship satisfaction over time, while relationship satisfaction predicts increases in growth beliefs over time. Identifying protective factors that prevent the typical deterioration of relationship satisfaction in long-term relationships is crucial for developing potential interventions to maintain relationship satisfaction.

## Supplemental Material

Supplemental Material - The role of relationship beliefs in predicting levels and changes of relationship satisfaction Supplemental Material for The role of relationship beliefs in predicting levels and changes of relationship satisfaction by Fabian Gander, Maximiliane Uhlich, Alex Christoph Traut, Marcelle Ariane Saameli, Janina Larissa Bühler, Rebekka Weidmann and Alexander Grob in European Journal of Personality.

## Notes

1. We decided to focus on actor effects in research question 4, since partner effects did not show to play a crucial role in research questions 1 to 3, and since the outcome was a one-item measure with limited variance (and reliability). For this purpose, we decided to simplify the model estimations.

2. Several earlier studies have used parts of the data from the CouPers project: Barbaro et al. (2021); Bühler et al. (2020); Bühler et al. (2021a); Francis et al. (2023); Gonzalez Avilés et al. (2021); Johnson et al. (2021); Mund et al. (2022); Nikitin et al. (2021); Reitz et al. (2022).

3. Participants were in a new relationship at the beginning of the study.

4. Although age and relationship duration were strongly correlated (*r* = .78), multicollinearity diagnostics were acceptable (all variance inflation factors <3) and we decided to include both as covariates.

5. In supplemental analyses, we also included the interaction between growth and destiny beliefs which did not predict levels or trajectories of relationship satisfaction, nor change any of the reported findings. Therefore, the interaction terms were excluded for not further complicating the model.

## References

[bibr1-08902070241240029] Anderson J. R. Van Ryzin M. J. Doherty W. J. (2010). Developmental trajectories of marital happiness in continuously married individuals: A group-based modeling approach. Journal of Family Psychology, 24(5), 587–596. 10.1037/a0020928 20954769

[bibr2-08902070241240029] Aust F. Barth M. (2022). Papaja: Prepare reproducible APA journal articles with R Markdown. https://github.com/crsh/papaja

[bibr3-08902070241240029] Barbaro N. Weidmann R. Burriss R. P. Wünsche J. Bühler J. L. Shackelford T. K. Grob A. (2021). The (bidirectional) associations between romantic attachment orientations and mate retention behavior in male-female romantic couples. Evolution and Human Behavior, 42(6), 497–506. 10.1016/j.evolhumbehav.2021.04.005

[bibr4-08902070241240029] Barton A. W. Lavner J. A. Stanley S. M. Johnson M. D. Rhoades G. K. (2020). “Will you complete this survey too?” Differences between individual versus dyadic samples in relationship research. Journal of Family Psychology, 34(2), 196–203. 10.1037/fam0000583 31380689 PMC7000299

[bibr5-08902070241240029] Beck A. T. (1970). Cognitive therapy: Nature and relation to behavior therapy. Behavior Therapy, 1(2), 184–200. 10.1016/S0005-7894(70)80030-2

[bibr6-08902070241240029] Bőthe B. Tóth-Király I. Demetrovics Z. Orosz G. (2017). The pervasive role of sex mindset: Beliefs about the malleability of sexual life is linked to higher levels of relationship satisfaction and sexual satisfaction and lower levels of problematic pornography use. Personality and Individual Differences, 117, 15–22. 10.1016/j.paid.2017.05.030

[bibr7-08902070241240029] Bühler J. L. Weidmann R. Wünsche J. Burriss R. P. Grob A. (2020). Daily responsiveness, expectations, and self–disclosure: How the average levels and within–person variability of three relationship components mediate personality–relationship transactions in romantic couples. European Journal of Personality, 34(3), 367–392. 10.1002/per.2255

[bibr8-08902070241240029] Bühler J. L. Krauss S. Orth U. (2021a). Development of relationship satisfaction across the life span: A systematic review and meta-analysis. Psychological Bulletin, 147(10), 1012. 10.1037/bul0000342 34928690

[bibr9-08902070241240029] Bühler J. L. Wrzus C. Weidmann R. Wünsche J. Burriss R. P. Grob A. (2021b). Hard-working in general but lazy at home? Generalized Big five traits and relationship-specific traits in romantic couples over time. Journal of Research in Personality, 92, 104087. 10.1016/j.jrp.2021.104087

[bibr10-08902070241240029] Burnette J. L. Franiuk R. (2010). Individual differences in implicit theories of relationships and partner fit: Predicting forgiveness in developing relationships. Personality and Individual Differences, 48(2), 144–148. 10.1016/j.paid.2009.09.011

[bibr86-9089020702419] Busseri M. A. Samani M. N. (2019). Lay theories for life satisfaction and the belief that life gets better and better. Journal of Happiness Studies, 20(5), 1647–1672. 10.1007/s10902-018-0016-x

[bibr11-08902070241240029] Butzer B. Campbell L. (2008). Adult attachment, sexual satisfaction, and relationship satisfaction: A study of married couples. Personal Relationships, 15(1), 141–154. 10.1111/j.1475-6811.2007.00189.x

[bibr12-08902070241240029] Chen Z. DeWall C. N. Poon K.-T. Chen E.-W. (2012). When destiny hurts: Implicit theories of relationships moderate aggressive responses to ostracism. Journal of Experimental Social Psychology, 48(5), 1029–1036. 10.1016/j.jesp.2012.04.002

[bibr13-08902070241240029] Clifton J. D. W. Baker J. D. Park C. L. Yaden D. B. Clifton A. B. W. Terni P. Miller J. L. Zeng G. Giorgi S. Schwartz H. A. Seligman M. E. P. (2019). Primal world beliefs. Psychological Assessment, 31(1), 82–99. 10.1037/pas0000639 30299119

[bibr14-08902070241240029] Cobb R. A. DeWall C. N. Lambert N. M. Fincham F. D. (2013). Implicit theories of relationships and close relationship violence: Does believing your relationship can grow relate to lower perpetration of violence? Personality and Social Psychology Bulletin, 39(3), 279–290. 10.1177/0146167212473159 23376887

[bibr82-9089020702416] Diener E. Emmons R. A. Larsen R. J. Griffin S. (1985). The satisfaction with life scale. Journal of Personality Assessment, 49(1), 71–75. 10.1207/s15327752jpa4901_13 16367493

[bibr100-08902070241240029] Dorrance-Hall E. Sharabi L. Roaché D. J. James-Hawkins L. Croft A. Alexopoulos C. Lamarche V. M. Uhlich M. Timmermans E. (2023). Needing space during lockdown: A test of relational turbulence theory in the context of conversations about physical and emotional space during the COVID-19 pandemic. Communication Research, 50(8), 943–964. 10.1177/00936502231174771

[bibr17-08902070241240029] DIW Berlin/SOEP . (2016). Soep 2016—erhebungsinstrumente 2016 (Welle 33) des Sozio-oekonomischen Panels: Personenfragebogen, Stichproben A-L3. (SOEP Survey Papers No. 345). Deutsches Institut für Wirtschaftsforschung (DIW). https://hdl.handle.net/10419/148433

[bibr19-08902070241240029] Dweck C. S. (1999). Self-theories: Their role in motivation, personality, and development. Psychology Press. 10.4324/9781315783048 2130257

[bibr20-08902070241240029] Erikson E. H. (1959). Identity and the life cycle. International Universities Press.

[bibr21-08902070241240029] Figueroa J. M. DeLuca Bishop H. K. Baker E. A. (2022). Using a socio-ecological framework to understand romantic relationship satisfaction among emerging adults during the COVID-19 pandemic. Emerging Adulthood, 10(6), 1561–1573. 10.1177/21676968221124266 38603198 PMC9434192

[bibr22-08902070241240029] Flora D. B. (2020). Your coefficient alpha is probably wrong, but which coefficient omega is right? A tutorial on using R to obtain better reliability estimates. Advances in Methods and Practices in Psychological Science, 3(4), 484–501. 10.1177/2515245920951747

[bibr23-08902070241240029] Fraley R. C. Heffernan M. E. Vicary A. M. Brumbaugh C. C. (2011). The experiences in close relationships—relationship structures questionnaire: A method for assessing attachment orientations across relationships. Psychological Assessment, 23(3), 615–625. 10.1037/a0022898 21443364

[bibr24-08902070241240029] Francis Z. Weidmann R. Bühler J. L. Burriss R. P. Wünsche J. Grob A. Job V. (2023). My willpower belief and yours: Investigating dyadic associations between willpower beliefs, social support, and relationship satisfaction in couples. European Journal of Personality. 10.1177/08902070231220416 PMC13021047 41909819

[bibr25-08902070241240029] Franiuk R. Cohen D. Pomerantz E. M. (2002). Implicit theories of relationships: Implications for relationship satisfaction and longevity. Personal Relationships, 9(4), 345–367. 10.1111/1475-6811.09401

[bibr26-08902070241240029] Franiuk R. Pomerantz E. M. Cohen D. (2004). The causal role of theories of relationships: Consequences for satisfaction and cognitive strategies. Personality and Social Psychology Bulletin, 30(11), 1494–1507. 10.1177/0146167204264894 15448312

[bibr27-08902070241240029] Frazier P. A. Cook S. W. (1993). Correlates of distress following heterosexual relationship dissolution. Journal of Social and Personal Relationships, 10(1), 55–67. 10.1177/0265407593101004

[bibr28-08902070241240029] Frick H. Chow F. Kuhn M. Mahoney M. Silge J. Wickham H. (2023). Rsample: General resampling infrastructure. https://CRAN.R-project.org/package=rsample

[bibr29-08902070241240029] Gander F. Wagner L. (2022). Character growth following collective life events? A study on perceived and measured changes in character strengths during the first wave of the COVID-19 pandemic. European Journal of Personality, 36(4), 466–482. 10.1177/08902070211040975 PMC13029417 41909813

[bibr30-08902070241240029] Gilford R. Bengtson V. (1979). Measuring marital satisfaction in three generations: Positive and negative dimensions. Journal of Marriage and Family, 41(2), 387–398. 10.2307/351705

[bibr83-9089020702417] Glaesmer H. Grande G. Braehler E. Roth M. (2011). The German version of the Satisfaction With Life Scale (SWLS). European Journal of Psychological Assessment, 27(2), 127–132. 10.1027/1015-5759/a000058

[bibr31-08902070241240029] Glenn N. D. (1990). Quantitative research on marital quality in the 1980s: A critical review. Journal of Marriage and Family, 52(4), 818–831. 10.2307/353304

[bibr32-08902070241240029] Goldberg L. R. (1993). The structure of phenotypic personality traits. American Psychologist, 48(1), 26–34. 10.1037/0003-066X.48.1.26 8427480

[bibr33-08902070241240029] Gonzalez Avilés T. Burriss R. P. Weidmann R. Bühler J. L. Wünsche J. Grob A. (2021). Committing to a romantic partner: Does attractiveness matter? A dyadic approach. Personality and Individual Differences, 176, 110765. 10.1016/j.paid.2021.110765

[bibr34-08902070241240029] Goodfriend W. Agnew C. R. (2008). Sunken costs and desired plans: Examining different types of investments in close relationships. Personality and Social Psychology Bulletin, 34(12), 1639–1652. 10.1177/0146167208323743 18779375

[bibr35-08902070241240029] Greif G. L. Deal K. H. (2012). The impact of divorce on friendships with couples and individuals. Journal of Divorce and Remarriage, 53(6), 421–435. 10.1080/10502556.2012.682894

[bibr36-08902070241240029] Hendrick S. S. (1988). A generic measure of relationship satisfaction. Journal of Marriage and Family, 50(1), 93–98. 10.2307/352430

[bibr37-08902070241240029] Holt-Lunstad J. Smith T. B. Layton J. B. (2010). Social relationships and mortality risk: A meta-analytic review. PLoS Medicine, 7(7), . 10.1371/journal.pmed.1000316 PMC2910600 20668659

[bibr38-08902070241240029] Iida M. Savord A. Ledermann T. (2023). Dyadic longitudinal models: A critical review. Personal Relationships, 30(2), 356–378. 10.1111/pere.12468

[bibr39-08902070241240029] Jackson J. B. Miller R. B. Oka M. Henry R. G. (2014). Gender differences in marital satisfaction: A meta-analysis. Journal of Marriage and Family, 76(1), 105–129. 10.1111/jomf.12077

[bibr40-08902070241240029] Joel S. Eastwick P. W. Allison C. J. Arriaga X. B. Baker Z. G. Bar-Kalifa E. Bergeron S. Birnbaum G. E. Brock R. L. Brumbaugh C. C. Carmichael C. L. Chen S. Clarke J. Cobb R. J. Coolsen M. K. Davis J. de Jong D. C. Debrot A. DeHaas E. C. Wolf S. (2020). Machine learning uncovers the most robust self-report predictors of relationship quality across 43 longitudinal couples studies. Proceedings of the National Academy of Sciences, 117(32), 19061–19071. 10.1073/pnas.1917036117 PMC7431040 32719123

[bibr41-08902070241240029] John O. P. Srivastava S. (1999). The Big Five trait taxonomy: History, measurement, and theoretical perspectives. In Pervin L. A. John O. P. (Eds.), Handbook of personality: Theory and research (pp. 102–138): The Guilford Press.

[bibr42-08902070241240029] Johnson M. D. Lavner J. A. Mund M. Zemp M. Stanley S. M. Neyer F. J. Impett E. A. Rhoades G. K. Bodenmann G. Weidmann R. Bühler J. L. Burriss R. P. Wünsche J. Grob A. (2021). Within-couple associations between communication and relationship satisfaction over time. Personality and Social Psychology Bulletin, 48(4), 01461672211016920. 10.1177/01461672211016920 PMC8915221 34027722

[bibr43-08902070241240029] Karney B. R. Bradbury T. N. (1995). The longitudinal course of marital quality and stability: A review of theory, methods, and research. Psychological Bulletin, 118(1), 3–34. 7644604 10.1037/0033-2909.118.1.3

[bibr44-08902070241240029] Kashy D. A. Donnellan M. B. (2008). Comparing MLM and SEM approaches to analyzing developmental dyadic data: Growth curve models of hostility in families. In Modeling dyadic and interdependent data in the developmental and behavioral sciences (pp. 165–190). Routledge/Taylor and Francis Group.

[bibr45-08902070241240029] Kenny D. A. Kashy D. A. Cook W. L. (2006). Dyadic data analysis. Guilford Press.

[bibr46-08902070241240029] Knee C. R. (1998). Implicit theories of relationships: Assessment and prediction of romantic relationship initiation, coping, and longevity. Journal of Personality and Social Psychology, 74(2), 360–370. 10.1037/0022-3514.74.2.360

[bibr47-08902070241240029] Knee C. R. Canevello A. (2006). Implicit theories of relationships and coping in romantic relationships. In Self and relationships: Connecting intrapersonal and interpersonal processes (pp. 160–176). The Guilford Press.

[bibr48-08902070241240029] Knee C. R. Nanayakkara A. Vietor N. A. Neighbors C. Patrick H. (2001). Implicit theories of relationships: Who cares if romantic partners are less than ideal? Personality and Social Psychology Bulletin, 27(7), 808–819. 10.1177/0146167201277004

[bibr49-08902070241240029] Knee C. R. Patrick H. Lonsbary C. (2003). Implicit theories of relationships: Orientations toward evaluation and cultivation. Personality and Social Psychology Review, 7(1), 41–55. 10.1207/S15327957PSPR0701_3 12584056

[bibr50-08902070241240029] Knee C. R. Patrick H. Vietor N. A. Neighbors C. (2004). Implicit theories of relationships: Moderators of the link between conflict and commitment. Personality and Social Psychology Bulletin, 30(5), 617–628. 10.1177/0146167203262853 15107161

[bibr52-08902070241240029] Le B. Dove N. L. Agnew C. R. Korn M. S. Mutso A. A. (2010). Predicting nonmarital romantic relationship dissolution: A meta-analytic synthesis. Personal Relationships, 17(3), 377–390. 10.1111/j.1475-6811.2010.01285.x

[bibr53-08902070241240029] Lüdecke D. (2022). sjPlot: Data visualization for statistics in social science. https://CRAN.R-project.org/package=sjPlot

[bibr54-08902070241240029] Mattingly B. A. McIntyre K. P. Knee C. R. Loving T. J. (2019). Implicit theories of relationships and self-expansion: Implications for relationship functioning. Journal of Social and Personal Relationships, 36(6), 1579–1599. 10.1177/0265407518768079

[bibr55-08902070241240029] Maxwell J. A. Muise A. MacDonald G. Day L. C. Rosen N. O. Impett E. A. (2017). How implicit theories of sexuality shape sexual and relationship well-being. Journal of Personality and Social Psychology, 112(2), 238. 10.1037/pspi0000078 27808534

[bibr56-08902070241240029] Mund M. Weidmann R. Wrzus C. Johnson M. D. Bühler J. L. Burriss R. P. Wünsche J. Grob A. (2022). Loneliness is associated with the subjective evaluation of but not daily dynamics in partner relationships. International Journal of Behavioral Development, 46(1), 28–38. 10.1177/0165025420951246

[bibr57-08902070241240029] Nikitin J. Wünsche J. Bühler J. L. Weidmann R. Burriss R. P. Grob A. (2021). Interdependence of approach and avoidance goals in romantic couples over days and months. The Journals of Gerontology: Serie Bibliographique, 76(7), 1251–1263. 10.1093/geronb/gbaa149 32882014

[bibr58-08902070241240029] Park Y. Impett E. A. MacDonald G. (2021). Generalizability of results from dyadic data: Participation of one versus two members of a romantic couple is associated with breakup likelihood. Personality and Social Psychology Bulletin, 47(2), 232–240. 10.1177/0146167220920167 32458730

[bibr59-08902070241240029] Pinheiro J. C. Bates D. M. (2000). Mixed-effects models in s and s-PLUS. Springer. 10.1007/b98882

[bibr60-08902070241240029] Rammstedt B. Danner D. (2016). Die Facettenstruktur des Big Five Inventory (BFI): Validierung fur die deutsche Adaptation des BFI. Diagnostica, 63(1), 70–84. 10.1026/0012-1924/a000161

[bibr61-08902070241240029] R Core Team . (2023). R: A language and environment for statistical computing. R Foundation for Statistical Computing. https://www.R-project.org/

[bibr62-08902070241240029] Reitz A. K. Weidmann R. Wünsche J. Bühler J. L. Burriss R. P. Grob A. (2022). In good times and in bad: A longitudinal analysis of the impact of bereavement on self-esteem and life satisfaction in couples. European Journal of Personality, 08902070211054896. 10.1177/08902070211054896

[bibr63-08902070241240029] Revelle W. (2022). psych: Procedures for psychological, psychometric, and personality research. Northwestern University. https://CRAN.R-project.org/package=psych

[bibr64-08902070241240029] Rhoades G. K. Kamp Dush C. M. Atkins D. C. Stanley S. M. Markman H. J. (2011). Breaking up is hard to do: The impact of unmarried relationship dissolution on mental health and life satisfaction. Journal of Family Psychology, 25(3), 366–374. 10.1037/a0023627 21517174 PMC3115386

[bibr65-08902070241240029] Roberts B. W. Wood D. (2006). Personality development in the context of the neo-socioanalytic model of personality. In Handbook of personality development (pp. 11–39). Lawrence Erlbaum Associates Publishers.

[bibr80-9089020702414] Rosenberg M. (1965). Society and the adolescent self-image. Princeton University Press.

[bibr67-08902070241240029] Rosseel Y. (2012). lavaan: An R package for structural equation modeling. Journal of Statistical Software, 48(2), 1–36. 10.18637/jss.v048.i02

[bibr68-08902070241240029] Sander J. Böcker S. (1993). Die deutsche Form der Relationship Assessment Scale (RAS): Eine kurze Skala zur Messung der Zufriedenheit in einer Partnerschaft. Diagnostica, 39(1), 55–62.

[bibr69-08902070241240029] Sbarra D. A. Emery R. E. (2005). The emotional sequelae of nonmarital relationship dissolution: Analysis of change and intraindividual variability over time. Personal Relationships, 12(2), 213–232. 10.1111/j.1350-4126.2005.00112.x

[bibr70-08902070241240029] Schneewind K. A. Wunderer E. Erkelenz M. (2004). Beziehungskompetenzen und beziehungsmuster in stabilen (langzeit-) ehen: ausgewahlte ergebnisse des Munchner DFG-projekts “was halt ehen zusammen? Zeitschrift fur Familienforschung, 16(3), 225–243.

[bibr71-08902070241240029] Soto C. J. (2019). How replicable are links between personality traits and consequential life outcomes? The life outcomes of personality replication project. Psychological Science, 30(5), 711–727. 10.1177/0956797619831612 30950321

[bibr72-08902070241240029] Sprecher S. Metts S. (1989). Dyadic longitudinal models: A critical review. Journal of Social and Personal Relationships, 6(4), 387–411. 10.1177/0265407589064001

[bibr73-08902070241240029] Srivastava S. McGonigal K. M. Richards J. M. Butler E. A. Gross J. J. (2006). Optimism in close relationships: How seeing things in a positive light makes them so. Journal of Personality and Social Psychology, 91(1), 143–153. 10.1037/0022-3514.91.1.143 16834485

[bibr74-08902070241240029] Thompson A. E. Capesius D. Kulibert D. Doyle R. A. (2020). Understanding infidelity forgiveness: An application of implicit theories of relationships. Journal of Relationships Research, 11, Article e2. 10.1017/jrr.2019.21

[bibr75-08902070241240029] Uhlich M. Nouri N. Jensen R. Meuwly N. Schoebi D. (2022). Associations of conflict frequency and sexual satisfaction with weekly relationship satisfaction in Iranian couples. Journal of Family Psychology, 36(1), 140–146. 10.1037/fam0000878 34081504

[bibr85-9089020702413] Uppot A. Raposo S. Rosen N. O. Corsini-Munt S. Balzarini R. Muise A. (2024). Responsiveness in the Face of Sexual Challenges: The Role of Sexual Growth and Destiny Beliefs. The Journal of Sex Research, 61(2), 228–245. 10.1080/00224499.2023.2175194 36787122

[bibr81-9089020702415] von Collani G. Herzberg P. Y. (2003). Eine revidierte Fassung der deutschsprachigen Skala zum Selbstwetgefühl von Rosenberg. [A revised version of the German adaptation of Rosenberg’s Self-Esteem Scale]. Zeitschrift Für Differentielle Und Diagnostische Psychologie, 24(1), 3–7. 10.1024/0170-1789.24.1.3

[bibr76-08902070241240029] Weidmann R. Chopik W. J. (2022). Romantic attachment, stress, and cognitive functioning in a large sample of middle-aged and older couples. Journal of Research in Personality, 98, Article 104233. 10.1016/j.jrp.2022.104233

[bibr77-08902070241240029] Weigel D. J. Lalasz C. B. Weiser D. A. (2016). Maintaining relationships: The role of implicit relationship theories and partner fit. Communication Reports, 29(1), 23–34. 10.1080/08934215.2015.1017653

[bibr78-08902070241240029] Weston S. Calaway R. (2022). Getting Started with doParallel and foreach. https://cran.r-project.org/web/packages/doParallel/vignettes/gettingstartedParallel.pdf

[bibr84-9089020702418] Wickham H. Averick M. Bryan J. Chang W. McGowan L. D. François R. Grolemund G. Hayes A. Henry L. Hester J. Kuhn M. Pedersen T. L. Miller E. Bache S. M. Müller K. Ooms J. Robinson D. Seidel D. P. Spinu V.… Yutani H. (2019). Welcome to the Tidyverse. Journal of Open Source Software, 4(43), 1686. 10.21105/joss.01686

[bibr79-08902070241240029] Yeager D. S. Dweck C. S. (2020). What can be learned from growth mindset controversies? American Psychologist, 75(9), 1269–1284. 10.1037/amp0000794 33382294 PMC8299535

