# Supplementary material for: The role of relationship beliefs in predicting levels and changes of relationship satisfaction
Source: Eur J Pers. 2024 Apr 9;39(1):105–21. doi: 10.1177/08902070241240029 (PMC13029515; doi:10.1177/08902070241240029)
Supplement: Supplemental Material - The role of relationship beliefs in predicting levels and changes of relationship satisfaction [file sj-pdf-1-erp-10.1177_08902070241240029.pdf]

Online Supplementary

Table S1

Sample Sizes, Means, and Standard Deviations of All Measures in the Study

	Partner A			Partner B		
	N	% women	% men	*N*	% women	% men
Gender	904	99.2	0.8	904	2.7	97.3
	N	*M*	*SD*	*N*	*M*	*SD*
Relationship Duration*	904	8.69	10.49	904	8.69	10.49
Age	904	31.99	13.49	904	33.99	14.08
Relationship Satisfaction T1	868	4.36	0.57	848	4.34	0.53
Relationship Satisfaction T2	693	4.34	0.57	651	4.26	0.57
Relationship Satisfaction T3	619	4.34	0.59	562	4.28	0.60
Relationship Satisfaction T4	505	4.28	0.64	451	4.29	0.61
Growth Beliefs T1	904	4.13	0.61	904	4.09	0.63
Growth Beliefs T2	761	4.24	0.57	727	4.16	0.62
Destiny Beliefs T1	904	2.87	0.84	904	2.90	0.82
Destiny Beliefs T2	761	4.24	0.57	727	4.16	0.62
Subjective trajectory of past relationship satisfaction						
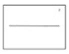	502	0.12	0.32	450	0.09	0.29
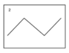	502	0.22	0.41	450	0.22	0.41
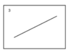	502	0.25	0.44	450	0.31	0.46
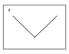	502	0.07	0.25	450	0.04	0.20
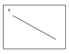	502	0.01	0.11	450	0.01	0.09
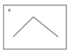	502	0.01	0.12	450	0.02	0.12
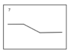	502	0.07	0.25	450	0.07	0.25
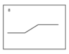	502	0.20	0.40	450	0.20	0.40
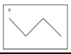	502	0.06	0.23	450	0.05	0.21
Subjective trajectory of future relationship satisfaction						
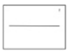	502	0.12	0.32	450	0.13	0.34
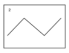	502	0.10	0.30	450	0.13	0.34
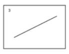	502	0.35	0.48	450	0.39	0.49
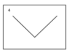	502	0.01	0.08	450	0.01	0.11
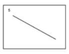	502	0.01	0.11	450	0.03	0.17
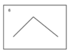	502	0.00	0.04	450	0.00	0.07
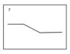	502	0.03	0.17	450	0.01	0.09
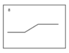	502	0.36	0.48	450	0.29	0.45

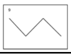	502	0.02	0.13	450	0.01	0.09
Further Individual Differences						
Neuroticism	904	3.15	0.77	904	2.56	0.71
Extraversion	904	3.63	0.75	904	3.41	0.76
Agreeableness	904	3.68	0.56	904	3.64	0.55
Conscientiousness	904	3.74	0.66	904	3.59	0.64
Openness	904	3.70	0.65	904	3.55	0.65
Self-Esteem	904	3.17	0.60	904	3.33	0.50
Life Satisfaction	904	3.79	0.80	903	3.77	0.75
Attachment Anxiety	904	2.30	1.31	904	2.16	1.17
Attachment Avoidance	904	1.97	0.92	904	2.19	0.93

Note. Demographic characteristics at the beginning of the study. * = Averaged across both partners. Subjective trajectories of past/future relationship satisfaction: 0 = not selected, 1 = selected.

Table S2

Zero-Order Correlations of All Measures in the Study

	Gender	Age	Relationship duration	Relationship Satisfaction T1	Relationship Satisfaction T2	Relationship Satisfaction T3	Relationship Satisfaction T4	Growth Beliefs T1	Growth Beliefs T2	Destiny Beliefs T1	Destiny Beliefs T2	Past Relationship Satisfaction Trajectory 1	Past Relationship Satisfaction Trajectory 2	Past Relationship Satisfaction Trajectory 3	Past Relationship Satisfaction Trajectory 8	Future Relationship Satisfaction Trajectory 1	Future Relationship Satisfaction Trajectory 2	Future Relationship Satisfaction Trajectory 3	Future Relationship Satisfaction Trajectory 8	Neuroticism	Extraversion	Agreeableness	Conscientiousness	Openness	Self-Esteem	Life Satisfaction	Attachment Anxiety
Age	.07																										
Relationship Duration	.00	.78																									
Relationship Satisfaction T1	-.03	-.08	-.10																								
Relationship Satisfaction T2	-.08	-.10	-.09	.79																							
Relationship Satisfaction T3	-.08	-.08	-.06	.74	.82																						
Relationship Satisfaction T4	-.02	-.05	-.04	.70	.75	.78																					
Growth Beliefs T1	-.03	-.10	.00	-.03	.01	.04	.03																				
Growth Beliefs T2	-.08	-.19	-.09	.08	.10	.12	.13	.59																			
Destiny Beliefs T1	.03	.09	.01	.08	.09	.04	.06	-.23	-.23																		
Destiny Beliefs T2	.03	.13	.07	.05	.07	.07	.09	-.23	-.26	.65																	
Past Relationship Satisfaction																											
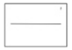	-.03	.03	.03	.07	.10	.11	.09	-.06	-.04	.00	.07																
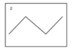	.00	-.06	-.01	-.15	-.15	-.15	-.07	.06	.08	-.03	-.09	-.18															
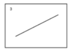	.05	.02	-.02	.30	.29	.32	.34	.03	.03	.01	.05	-.21	-.33														

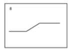	.00	.07	.05	.05	.06	.06	.10	-.04	-.02	.02	.02	-.17	-.26	-.31													
Future Relationship Satisfaction																											
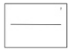	.03	.19	.17	.02	.02	.02	.04	-.09	-.07	.00	.03	.35	-.15	-.12	.04												
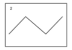	.04	-.05	-.05	-.12	-.12	-.10	-.10	.04	.05	-.02	-.06	-.07	.18	-.10	-.06	-.13											
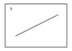	.03	-.05	-.06	.26	.26	.27	.30	.04	.06	.09	.07	-.11	-.05	.45	-.12	-.29	-.27										
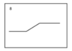	-.08	-.06	-.04	-.06	-.05	-.06	-.03	.01	.00	-.04	-.03	-.09	.08	-.23	.17	-.26	-.25	-.53									
Neuroticism	-.36	-.14	-.09	-.15	-.13	-.13	-.18	.06	.04	.00	-.01	-.01	.09	-.10	-.07	-.08	.01	-.02	.04								
Extraversion	-.14	.08	.05	.08	.09	.09	.13	.01	.05	.01	.01	-.05	-.03	.07	.04	-.03	.06	-.02	.02	-.16							
Agreeableness	-.05	.11	.10	.20	.17	.18	.17	.02	.04	-.09	-.08	.05	-.05	.07	.01	.03	-.05	.04	.00	-.30	.08						
Conscientiousness	-.11	.24	.21	.09	.06	.08	.05	.01	.03	.08	.06	.06	-.06	.05	.01	.05	-.02	.02	-.03	-.14	.16	.12					
Openness	-.12	.08	.03	.01	.03	.04	.03	.04	.03	-.06	-.08	-.08	.02	.01	.01	-.08	.03	-.01	.04	.00	.26	.08	.03				
Self-Esteem	.15	.16	.11	.24	.19	.19	.24	-.04	-.01	.02	.00	.03	-.04	.10	.03	.08	-.02	.02	-.03	-.56	.33	.21	.29	.10			
Life Satisfaction	-.02	.09	.11	.37	.33	.34	.37	.00	.04	.01	.02	.06	-.16	.15	.08	.09	-.06	.07	-.04	-.35	.27	.23	.24	.01	.58		
Attachment Anxiety	-.06	-.08	-.10	-.33	-.28	-.30	-.34	.03	-.01	.04	.05	-.09	.09	-.16	-.04	-.07	.04	-.08	.03	.28	-.13	-.19	-.11	-.03	-.37	-.30	
Attachment Avoidance	.14	.06	.04	-.49	-.45	-.42	-.39	-.08	-.14	.02	.04	-.04	.09	-.15	-.03	.00	.09	-.13	.01	.03	-.13	-.17	-.05	-.03	-.15	-.25	.34

Table S3

Measurement Invariance of Relationship Satisfaction Across Time

Invariance Level	χ^2	*df*	CFI	TLI	RMSEA	95 % CI	SRMR
Configural	1,183.01	604	.977	.971	.033	[.030, .036]	.038
Metric	1,263.63	646	.976	.972	.033	[.030, .036]	.045
Scalar	1,384.01	688	.973	.970	.034	[.031, .036]	.047

Note. *df* = degrees of freedom; CFI = Comparative fit index; TLI = Tucker-Lewis index; RMSEA = Root mean square error of approximation; SRMR = Standardized root mean square residual.

Table S4

Multilevel Model Results for Relationship Beliefs, Time, and Relationship Duration, and Their Interaction on Relationship Satisfaction

	b	95% CI	*t*	*df*	*p*
Fixed Effects					
Intercept	-.08	[-0.14, -0.02]	-2.59	4,254	.010
Age	.02	[-0.04, 0.07]	0.56	4,254	.575
Gender	-.05	[-0.08, -0.01]	-2.82	4,254	.005
Duration	-.12	[-0.21, -0.04]	-3.00	900	.003
Time	-.11	[-0.14, -0.08]	-7.18	4,254	< .001
Growth Beliefs (actor effect)	.02	[-0.03, 0.06]	0.85	4,254	.396
Destiny Beliefs (actor effect)	.06	[0.01, 0.10]	2.59	4,254	.010
Growth Beliefs (partner effect)	-.02	[-0.07, 0.02]	-1.07	4,254	.283
Destiny Beliefs (partner effect)	.03	[-0.01, 0.07]	1.31	4,254	.190
Growth Beliefs (actor × partner effect)	.08	[0.02, 0.14]	2.78	900	.006
Destiny Beliefs (actor × partner effect)	-.01	[-0.06, 0.05]	-0.22	900	.826
Time × Growth Beliefs (actor effect)	.03	[0.00, 0.05]	2.35	4,254	.019
Time × Destiny Beliefs (actor effect)	.00	[-0.02, 0.03]	0.17	4,254	.862
Time × Growth Beliefs (partner effect)	.00	[-0.03, 0.02]	-0.37	4,254	.712
Time × Destiny Beliefs (partner effect)	-.02	[-0.04, 0.00]	-1.56	4,254	.119
Time × Growth Beliefs (actor × partner effect)	.02	[-0.01, 0.04]	1.17	4,254	.243
Time × Destiny Beliefs (actor × partner effect)	-.03	[-0.06, 0.00]	-2.17	4,254	.030
Duration × Growth Beliefs (actor effect)	.02	[-0.02, 0.06]	0.98	4,254	.328
Duration × Destiny Beliefs (actor effect)	.03	[-0.01, 0.07]	1.42	4,254	.155
Duration × Growth Beliefs (partner effect)	.02	[-0.02, 0.06]	0.95	4,254	.341
Duration × Destiny Beliefs (partner effect)	.01	[-0.03, 0.05]	0.41	4,254	.679
Duration × Growth Beliefs (actor × partner effect)	-.01	[-0.07, 0.04]	-0.53	4,254	.596
Duration × Destiny Beliefs (actor × partner effect)	.03	[-0.02, 0.08]	1.30	4,254	.193
Gender × Time	.00	[-0.02, 0.02]	-0.19	4,254	.852
Gender × Duration	.06	[0.03, 0.09]	4.17	4,254	< .001
Gender × Growth Beliefs (actor effect)	.07	[0.03, 0.11]	3.62	4,254	< .001
Gender x Destiny Beliefs (actor effect)	.03	[0.00, 0.07]	1.79	4,254	.074
Gender × Growth Beliefs (partner effect)	-.04	[-0.08, 0.00]	-2.03	4,254	.043
Gender × Destiny Beliefs (partner effect)	-.01	[-0.05, 0.03]	-0.59	4,254	.553
Gender × Growth Beliefs (actor × partner effect)	.00	[-0.03, 0.03]	0.12	4,254	.907
Gender × Destiny Beliefs (actor × partner effect)	-.02	[-0.04, 0.01]	-1.11	4,254	.267
Gender × Time × Growth Beliefs (actor effect)	.01	[-0.01, 0.04]	0.92	4,254	.359
Gender × Time × Destiny Beliefs (actor effect)	.02	[-0.01, 0.04]	1.22	4,254	.222
Gender × Time × Growth Beliefs (partner effect)	.00	[-0.03, 0.02]	-0.17	4,254	.865
Gender × Time × Destiny Beliefs (partner effect)	.00	[-0.02, 0.03]	0.33	4,254	.745
Gender × Time × Growth Beliefs (actor × partner effect)	.00	[-0.04, 0.04]	-0.03	4,254	.977
Gender × Time × Destiny Beliefs (actor × partner effect)	.02	[-0.02, 0.06]	1.14	4,254	.253
Gender × Duration × Growth Beliefs (actor effect)	.00	[-0.04, 0.05]	0.05	4,254	.959
Gender × Duration × Destiny Beliefs (actor effect)	-.05	[-0.09, -0.01]	-2.34	4,254	.019
Gender × Duration × Growth Beliefs (partner effect)	.00	[-0.02, 0.02]	0.07	4,254	.945

	b	95% CI	*t*	*df*	*p*
Gender × Duration × Destiny Beliefs (partner effect)	.00	[-0.01, 0.02]	0.48	4,254	.629
Gender × Duration × Growth Beliefs (actor × partner effect)	-.01	[-0.04, 0.02]	-0.62	4,254	.532
Gender × Duration × Destiny Beliefs (actor × partner effect)	.00	[-0.03, 0.02]	-0.08	4,254	.932
Random Effects					
SD intercept women	.94				
SD intercept men	.89				
SD slope women	.26				
SD slope men	.26				
SD residual	.47				

Note. Age = Age in decades (grand mean centered), Duration = Relationship duration in decades (grand mean centered), Time = Time in years. Gender: -1 = women, +1 = men. All other variables are *z*-standardized.

Table S5

Multilevel Model Results for Relationship Beliefs, Time, and Relationship Duration, and Their Interaction on Relationship Satisfaction, Corrected for Personality, Attachment, Life Satisfaction and Self-Esteem

	β	95% CI	*t*	*df*	*p*
Fixed Effects					
Intercept	-.09	[-0.14, -0.04]	-3.65	4,226	< .001
Age	.00	[-0.05, 0.05]	-0.06	4,226	.955
Gender	-.02	[-0.06, 0.02]	-1.07	4,226	.283
Duration	-.13	[-0.20, -0.06]	-3.76	900	< .001
Time	-.11	[-0.14, -0.08]	-7.04	4,226	< .001
Growth Beliefs (actor effect)	-.01	[-0.05, 0.02]	-0.69	4,226	.488
Destiny Beliefs (actor effect)	.05	[0.01, 0.09]	2.69	4,226	.007
Growth Beliefs (partner effect)	-.02	[-0.06, 0.02]	-1.01	4,226	.311
Destiny Beliefs (partner effect)	.03	[0.00, 0.07]	1.80	4,226	.072
Growth Beliefs (actor × partner effect)	.06	[0.02, 0.11]	2.66	900	.008
Destiny Beliefs (actor × partner effect)	-.02	[-0.06, 0.03]	-0.72	900	.471
Neuroticism	-.04	[-0.08, 0.00]	-1.88	4,226	.060
Extraversion	.01	[-0.02, 0.05]	0.76	4,226	.448
Agreeableness	.04	[0.00, 0.07]	2.07	4,226	.038
Conscientiousness	.01	[-0.03, 0.04]	0.47	4,226	.637
Openness	-.01	[-0.05, 0.02]	-0.74	4,226	.462
Self-Esteem	-.04	[-0.09, 0.01]	-1.75	4,226	.080
Life Satisfaction	.20	[0.16, 0.25]	8.71	4,226	< .001
Attachment Anxiety	-.09	[-0.13, -0.05]	-4.93	4,226	< .001
Attachment Avoidance	-.29	[-0.32, -0.25]	-14.72	4,226	< .001
Time × Growth Beliefs (actor effect)	.03	[0.01, 0.05]	2.39	4,226	.017
Time × Destiny Beliefs (actor effect)	.00	[-0.02, 0.03]	0.11	4,226	.916
Time × Growth Beliefs (partner effect)	.00	[-0.03, 0.02]	-0.11	4,226	.910
Time × Destiny Beliefs (partner effect)	-.02	[-0.04, 0.01]	-1.49	4,226	.137
Time × Growth Beliefs (actor × partner effect)	.01	[-0.01, 0.04]	1.05	4,226	.295
Time × Destiny Beliefs (actor × partner effect)	-.03	[-0.06, -0.01]	-2.34	4,226	.019
Time × Neuroticism	.00	[-0.03, 0.02]	-0.30	4,226	.762
Time × Extraversion	.02	[0.00, 0.05]	2.01	4,226	.044
Time × Agreeableness	.00	[-0.02, 0.03]	0.41	4,226	.682
Time × Conscientiousness	.00	[-0.02, 0.02]	0.10	4,226	.919
Time × Openness	-.01	[-0.03, 0.02]	-0.61	4,226	.539
Time × Self-Esteem	-.04	[-0.07, 0.00]	-2.23	4,226	.026
Time × Life Satisfaction	.03	[0.00, 0.06]	1.84	4,226	.065
Time × Attachment Anxiety	-.02	[-0.04, 0.01]	-1.29	4,226	.197
Time × Attachment Avoidance	.02	[0.00, 0.05]	1.89	4,226	.058
Duration × Growth Beliefs (actor effect)	.00	[-0.04, 0.03]	-0.14	4,226	.886
Duration × Destiny Beliefs (actor effect)	.01	[-0.02, 0.04]	0.71	4,226	.481
Duration × Growth Beliefs (partner effect)	-.01	[-0.04, 0.03]	-0.39	4,226	.699

	β	95% CI	*t*	*df*	*p*
Duration × Destiny Beliefs (partner effect)	-.01	[-0.04, 0.02]	-0.62	4,226	.535
Duration × Growth Beliefs (actor × partner effect)	-.01	[-0.05, 0.03]	-0.36	4,226	.722
Duration × Destiny Beliefs (actor × partner effect)	.03	[-0.01, 0.07]	1.41	4,226	.159
Duration × Neuroticism	.03	[-0.01, 0.07]	1.56	4,226	.119
Duration × Extraversion	.01	[-0.02, 0.05]	0.75	4,226	.456
Duration × Agreeableness	.02	[-0.02, 0.05]	0.84	4,226	.402
Duration × Conscientiousness	.02	[-0.02, 0.07]	1.15	4,226	.251
Duration × Openness	-.03	[-0.06, 0.01]	-1.58	4,226	.113
Duration × Self-Esteem	.00	[-0.05, 0.05]	0.03	4,226	.979
Duration × Life Satisfaction	.04	[-0.01, 0.09]	1.56	4,226	.119
Duration × Attachment Anxiety	-.04	[-0.08, 0.00]	-1.84	4,226	.066
Duration × Attachment Avoidance	.02	[-0.02, 0.05]	0.89	4,226	.372
Gender × Time	.00	[-0.02, 0.03]	0.27	4,226	.790
Gender × Duration	.08	[0.05, 0.11]	4.77	4,226	< .001
Gender × Growth Beliefs (actor effect)	.03	[-0.01, 0.06]	1.43	4,226	.151
Gender x Destiny Beliefs (actor effect)	.01	[-0.02, 0.05]	0.67	4,226	.504
Gender × Growth Beliefs (partner effect)	.00	[-0.04, 0.03]	-0.18	4,226	.857
Gender × Destiny Beliefs (partner effect)	.00	[-0.03, 0.04]	0.27	4,226	.784
Gender × Growth Beliefs (actor × partner effect)	.01	[-0.02, 0.04]	0.51	4,226	.610
Gender × Destiny Beliefs (actor × partner effect)	-.02	[-0.04, 0.01]	-1.18	4,226	.240
Gender × Time × Growth Beliefs (actor effect)	.01	[-0.01, 0.04]	0.99	4,226	.323
Gender × Time × Destiny Beliefs (actor effect)	.02	[-0.01, 0.04]	1.21	4,226	.226
Gender × Time × Growth Beliefs (partner effect)	.00	[-0.03, 0.02]	-0.34	4,226	.733
Gender × Time × Destiny Beliefs (partner effect)	.00	[-0.02, 0.02]	0.03	4,226	.976
Gender × Time × Growth Beliefs (actor × partner effect)	.00	[-0.02, 0.02]	0.15	4,226	.880
Gender × Time x Destiny Beliefs (actor × partner effect)	.00	[-0.01, 0.02]	0.37	4,226	.713
Gender × Duration × Growth Beliefs (actor effect)	.00	[-0.04, 0.04]	-0.10	4,226	.920
Gender × Duration × Destiny Beliefs (actor effect)	.01	[-0.03, 0.04]	0.39	4,226	.696
Gender × Duration × Growth Beliefs (partner effect)	.02	[-0.02, 0.06]	1.04	4,226	.300
Gender × Duration × Destiny Beliefs (partner effect)	-.01	[-0.05, 0.02]	-0.82	4,226	.411
Gender × Duration × Growth Beliefs (actor × partner effect)	-.01	[-0.04, 0.02]	-0.57	4,226	.568
Gender × Duration × Destiny Beliefs (actor × partner effect)	-.01	[-0.03, 0.02]	-0.68	4,226	.498
Random Effects					
SD intercept women	.75				
SD intercept men	.72				
SD slope women	.25				
SD slope men	.26				
SD residual	.47				

Note. Age = Age in decades (grand mean centered), Duration = Relationship duration in decades (grand mean centered), Time = Time in years. Gender: -1 = women, +1 = men. All other variables are *z*-standardized.

Table S6

Multilevel Model Results for Relationship Satisfaction, Time, and Relationship Duration, and Their Interaction on Growth Beliefs

	b	95% CI	*t*	*df*	*p*
Fixed Effects					
Intercept	.04	[-0.01, 0.10]	1.62	2,192	.106
Age	-.21	[-0.26, -0.15]	-7.47	2,192	< .001
Gender	-.03	[-0.08, 0.01]	-1.53	2,192	.127
Duration	.16	[0.08, 0.24]	4.16	818	< .001
Time	.16	[0.10, 0.21]	5.79	2,192	< .001
Satisfaction (actor effect)	.03	[-0.02, 0.08]	1.06	2,192	.289
Satisfaction (partner effect)	-.05	[-0.10, 0.00]	-1.92	2,192	.055
Satisfaction (actor × partner effect)	-.04	[-0.09, 0.00]	-1.86	818	.063
Time × Satisfaction (actor effect)	.07	[0.01, 0.13]	2.28	2,192	.023
Time × Satisfaction (partner effect)	.05	[-0.01, 0.11]	1.64	2,192	.101
Time × Satisfaction (actor × partner effect)	-.02	[-0.06, 0.03]	-0.67	2,192	.501
Duration × Satisfaction (actor effect)	.02	[-0.03, 0.07]	0.68	2,192	.498
Duration × Satisfaction (partner effect)	.02	[-0.03, 0.07]	0.75	2,192	.453
Duration × Satisfaction (actor × partner effect)	.02	[-0.01, 0.06]	1.33	2,192	.185
Gender × Time	-.03	[-0.08, 0.02]	-1.17	2,192	.243
Gender × Duration	.02	[-0.02, 0.06]	0.85	2,192	.396
Gender × Satisfaction (actor effect)	.07	[0.01, 0.12]	2.40	2,192	.017
Gender × Satisfaction (partner effect)	-.06	[-0.11, 0.00]	-2.06	2,192	.039
Gender × Satisfaction (actor × partner effect)	.01	[-0.03, 0.05]	0.41	2,192	.678
Gender × Time × Satisfaction (actor effect)	-.02	[-0.08, 0.04]	-0.58	2,192	.563
Gender × Time × Satisfaction (partner effect)	-.02	[-0.08, 0.04]	-0.78	2,192	.434
Gender × Duration× Satisfaction (actor × partner effect)	-.01	[-0.06, 0.03]	-0.61	2,192	.541
Gender × Duration× Satisfaction (actor effect)	.00	[-0.06, 0.06]	-0.04	2,192	.971
Gender × Duration× Satisfaction (partner effect)	.00	[-0.05, 0.06]	0.11	2,192	.911
Gender × Duration× Satisfaction (actor × partner effect)	.01	[-0.02, 0.04]	0.56	2,192	.579
Random Effects					
SD intercept women	.70				
SD intercept men	.78				
SD residual	.63				

Note. Age = Age in decades (grand mean centered), Duration = Relationship duration in decades (grand mean centered), Time = Time in years. Gender: -1 = women, +1 = men. All other variables are *z*-standardized.

Table S7

Multilevel Model Results for Relationship Satisfaction, Time, and Relationship Duration, and Their Interaction on Destiny Beliefs

	b	95% CI	*t*	*df*	*p*
Fixed Effects					
Intercept	-.03	[-0.09, 0.02]	-1.08	2,192	.278
Age	.15	[0.10, 0.21]	5.41	2,192	< .001
Gender	.02	[-0.02, 0.07]	1.04	2,192	.299
Duration	-.12	[-0.19, -0.04]	-2.94	818	.003
Time	-.09	[-0.14, -0.04]	-3.72	2,192	< .001
Satisfaction (actor effect)	.06	[0.01, 0.11]	2.38	2,192	.017
Satisfaction (partner effect)	.06	[0.01, 0.12]	2.46	2,192	.014
Satisfaction (actor × partner effect)	.04	[-0.01, 0.09]	1.68	818	.093
Time × Satisfaction (actor effect)	-.03	[-0.08, 0.03]	-0.97	2,192	.332
Time × Satisfaction (partner effect)	.01	[-0.05, 0.06]	0.22	2,192	.825
Time × Satisfaction (actor × partner effect)	.00	[-0.04, 0.04]	-0.01	2,192	.989
Duration × Satisfaction (actor effect)	.04	[-0.01, 0.10]	1.67	2,192	.095
Duration × Satisfaction (partner effect)	.01	[-0.04, 0.06]	0.41	2,192	.683
Duration × Satisfaction (actor × partner effect)	.01	[-0.02, 0.05]	0.57	2,192	.567
Gender × Time	-.01	[-0.06, 0.04]	-0.20	2,192	.838
Gender × Duration	.04	[0.00, 0.08]	1.87	2,192	.061
Gender × Satisfaction (actor effect)	.01	[-0.04, 0.07]	0.48	2,192	.630
Gender × Satisfaction (partner effect)	-.02	[-0.08, 0.04]	-0.70	2,192	.484
Gender × Satisfaction (actor × partner effect)	.00	[-0.04, 0.04]	0.07	2,192	.945
Gender × Time × Satisfaction (actor effect)	.03	[-0.02, 0.09]	1.13	2,192	.257
Gender × Time × Satisfaction (partner effect)	-.03	[-0.09, 0.02]	-1.15	2,192	.249
Gender × Duration× Satisfaction (actor × partner effect)	.01	[-0.04, 0.05]	0.37	2,192	.715
Gender × Duration× Satisfaction (actor effect)	.02	[-0.03, 0.08]	0.79	2,192	.427
Gender × Duration× Satisfaction (partner effect)	.00	[-0.06, 0.06]	0.02	2,192	.981
Gender × Duration× Satisfaction (actor × partner effect)	.00	[-0.03, 0.03]	0.06	2,192	.949
Random Effects					
SD intercept women	.78				
SD intercept men	.73				
SD residual	.62				

Note. Age = Age in decades (grand mean centered), Duration = Relationship duration in decades (grand mean centered), Time = Time in years. Gender: -1 = women, +1 = men. All other variables are *z*-standardized.
